# Revealing Phosphorus Nitrides up to the Megabar Regime: Synthesis of α′‐P_3_N_5,_ δ‐P_3_N_5_ and PN_2_

**DOI:** 10.1002/chem.202201998

**Published:** 2022-10-17

**Authors:** Dominique Laniel, Florian Trybel, Adrien Néri, Yuqing Yin, Andrey Aslandukov, Timofey Fedotenko, Saiana Khandarkhaeva, Ferenc Tasnádi, Stella Chariton, Carlotta Giacobbe, Eleanor Lawrence Bright, Michael Hanfland, Vitali Prakapenka, Wolfgang Schnick, Igor A. Abrikosov, Leonid Dubrovinsky, Natalia Dubrovinskaia

**Affiliations:** ^1^ Material Physics and Technology at Extreme Conditions Laboratory of Crystallography University of Bayreuth 95440 Bayreuth Germany; ^2^ Centre for Science at Extreme Conditions and School of Physics and Astronomy University of Edinburgh EH9 3FD Edinburgh UK; ^3^ Department of Physics Chemistry and Biology (IFM) Linköping University 58183 Linköping Sweden; ^4^ Bayerisches Geoinstitut University of Bayreuth 95440 Bayreuth Germany; ^5^ State Key Laboratory of Crystal Materials Shandong University Jinan 250100 P. R. China; ^6^ Deutsches Elektronen-Synchrotron Notkestr. 85 22607 Hamburg Germany; ^7^ Center for Advanced Radiation Sources University of Chicago Chicago IL 60637 USA; ^8^ European Synchrotron Radiation Facility B.P. 220 38043 Grenoble Cedex France; ^9^ Department of Chemistry University of Munich (LMU) Butenandtstrasse 5–13 81377 Munich Germany

**Keywords:** high-pressure high-temperature synthesis, non-metal nitrides, PN_6_ octahedra, synchrotron single-crystal X-ray diffraction, ultra-incompressibility

## Abstract

Non‐metal nitrides are an exciting field of chemistry, featuring a significant number of compounds that can possess outstanding material properties. These properties mainly rely on maximizing the number of strong covalent bonds, with crosslinked XN_6_ octahedra frameworks being particularly attractive. In this study, the phosphorus–nitrogen system was studied up to 137 GPa in laser‐heated diamond anvil cells, and three previously unobserved phases were synthesized and characterized by single‐crystal X‐ray diffraction, Raman spectroscopy measurements and density functional theory calculations. δ‐P_3_N_5_ and PN_2_ were found to form at 72 and 134 GPa, respectively, and both feature dense 3D networks of the so far elusive PN_6_ units. The two compounds are ultra‐incompressible, having a bulk modulus of *K*_0_=322 GPa for δ‐P_3_N_5_ and 339 GPa for PN_2_. Upon decompression below 7 GPa, δ‐P_3_N_5_ undergoes a transformation into a novel α′‐P_3_N_5_ solid, stable at ambient conditions, that has a unique structure type based on PN_4_ tetrahedra. The formation of α′‐P_3_N_5_ underlines that a phase space otherwise inaccessible can be explored through materials formed under high pressure.

## Graphical abstract

**Phase in, phase out**: Investigations of the phosphorus–nitrogen system in a laser‐heated diamond anvil cell resulted in the synthesis of new δ‐P_3_N_5_ and PN_2_ phases at 72 and 134 GPa, respectively. Their structures are composed of PN_6_ octahedra. Upon decompression, δ‐P_3_N_5_ transforms into the novel α′‐P_3_N_5_ solid that is stable under ambient conditions.

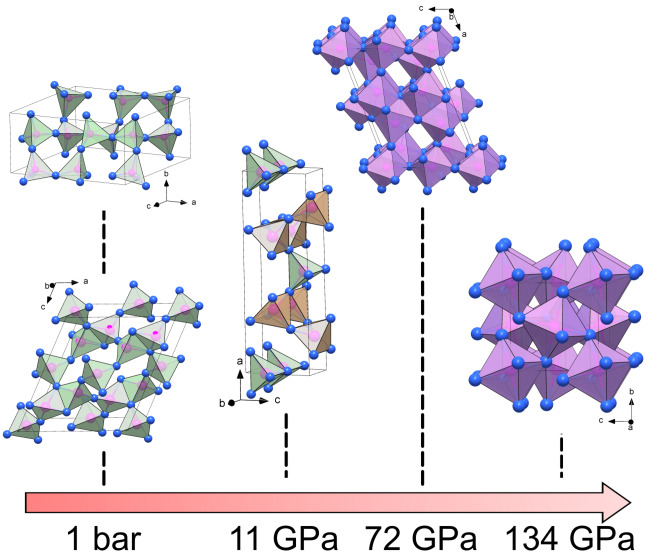

## Introduction

Non‐metal nitrides are prone to form dense and highly crosslinked networks made up of strongly bound alternating X and N atoms (X being a non‐metal). These solids are highly sought‐after for their exceptional materials properties, such as incompressibility and mechanical hardness, combined with a tendency of having high thermal stability, photocatalytic activity, chemical inertness and a wide bandgap with optoelectrical properties.[^1^, ^2^, ^3^, ^4^, ^5^, ^6^, ^7^] Prominent examples of binary non‐metal nitrides are the BN polymorphs (c‐BN, h‐BN),[^4^, ^8^] Si_3_N_4_,[^2^, ^9^] and diverse triazine‐ and heptazine‐based carbon nitrides.[^6^, ^10^, ^11^, ^12^] The structural chemistry of these compounds enables their impressive elastic properties through compact polyhedral arrangements, typically XN_4_ tetrahedra with strong covalent X−N bonds. For non‐metal elements capable of forming XN_5_ and XN_6_ polyhedra under high pressure, these can result in a significant hike in incompressibility, as demonstrated in the case of β‐Si_3_N_4_ (*K*_0_=∼237 GPa)^[13]^→γ‐Si_3_N_4_ (*K*_0_=∼300 GPa)^[9]^ and expected for α‐P_3_N_5_ (*K*_0_=134 GPa)^[14]^→V_3_O_5_‐type P_3_N_5_ (*K*_0_=303 GPa).^[14]^ At very high pressure, this can also be accompanied by a simultaneous change of stoichiometry and the formation of pernitride N_2_^x−^ units, such as in γ‐Si_3_N_4_→SiN_2_,^[15]^ β‐Ge_3_N_4_→GeN_2_,^[15]^ etc. and remarkably, these pressure‐formed higher coordinated compounds are often found recoverable to ambient conditions.

While transformation from XN_4_ to XN_6_ units was already observed in binary group 14 element nitrides (SiN_2_, GeN_2_, SnN_2_)^[15]^ and a chalcogen nitride (SN_2_) produced under pressure,^[16]^ XN_6_ octahedra are still eluding binary pnictogen nitrides.[^17^, ^18^, ^19^] This gap in our empirical understanding of non‐metal nitrides is especially striking given the significant efforts that were devoted to their synthesis, particularly for binary phosphorus nitrides. Still, the many experimental studies,[^18^, ^19^, ^20^, ^21^] supported by theoretical calculations,[^14^, ^22^, ^23^] amounted to the synthesis of three P_3_N_5_ polymorphs, namely α‐ and β‐P_3_N_5_, both formed at near ambient conditions,[^18^, ^20^] and γ‐P_3_N_5_ produced at 11 GPa and 1500 K.^[19]^ The exact crystal structure of β‐P_3_N_5_ is still unknown.[^18^, ^20^] α‐P_3_N_5_ can be represented by the Niggli formula^[24]^ ${}_{\infty }^{3}\left[P_{3}^{\left[4\right]}N_{3}^{\left[2\right]}N_{2}^{\left[3\right]}\right]$ – where the coordination number of a given atom is provided in superscripted square brackets (i. e., $P_{3}^{\left[4\right]}$ represents three phosphorus atoms each fourfold coordinated) and the dimensionality of the network is given by the number of dimensions, in superscript, in which the structural unit has an infinite extension (i. e., for the infinite polymeric 3D α‐P_3_N_5_, it is ${}_{\infty }{}^{3}\left[\cdots \right]$ ). α‐P_3_N_5_ features corner‐ and edge‐sharing PN_4_ units, whereas the pressure‐formed γ‐P_3_N_5_ contains both PN_4_ and PN_5_ polyhedra according to ${}_{\infty }^{3}\left[P_{1}^{\left[4\right]}P_{2}^{\left[5\right]}N_{1}^{\left[2\right]}N_{4}^{\left[3\right]}\right]$ . All these phosphorus nitrides are composed solely of heteroatomic P−N bonds. Further endeavours to produce a binary phosphorus nitride with PN_6_ octahedra by compressing γ‐P_3_N_5_ to 80 GPa did not result in any phase transformation, although laser‐heating γ‐P_3_N_5_ to 2000 K between 67 and 70 GPa resulted in the appearance of new Raman modes originating from a compound with an unsolved structure.^[21]^ Outside the substance class of nitrides, the existence of PN_6_ units has been confirmed in the molecular hexaazidophosphate anion.^[25]^

Only recently has the existence of PN_6_ units been confirmed in pressure‐synthesized ternary phosphorus nitrides^[26]^ β‐BP_3_N_6_ ^[27]^ and spinel‐type BeP_2_N_4_.^[28]^ Moreover, theoretical calculations support the formation of phosphorus nitrides with PN_6_ octahedra, with kyanite^[23]^ and V_3_O_5_ ^[22]^ as structure candidates being most likely. Importantly, these calculations also reveal that compositions other than P_3_N_5_ might be stable under pressure, namely PN_2_ and PN_3_. Both of the latter are expected to contain the elusive PN_6_ units as well.^[22]^ As such, there are substantial reasons to believe that further experiments on the P−N system could contribute to completing the more than 25‐year hunt for a binary pnictogen nitride solid with XN_6_ octahedra, finally getting back in line with group 14 and 16 (chalcogen) nitrides.

## Results and Discussion

**Figure 1:**
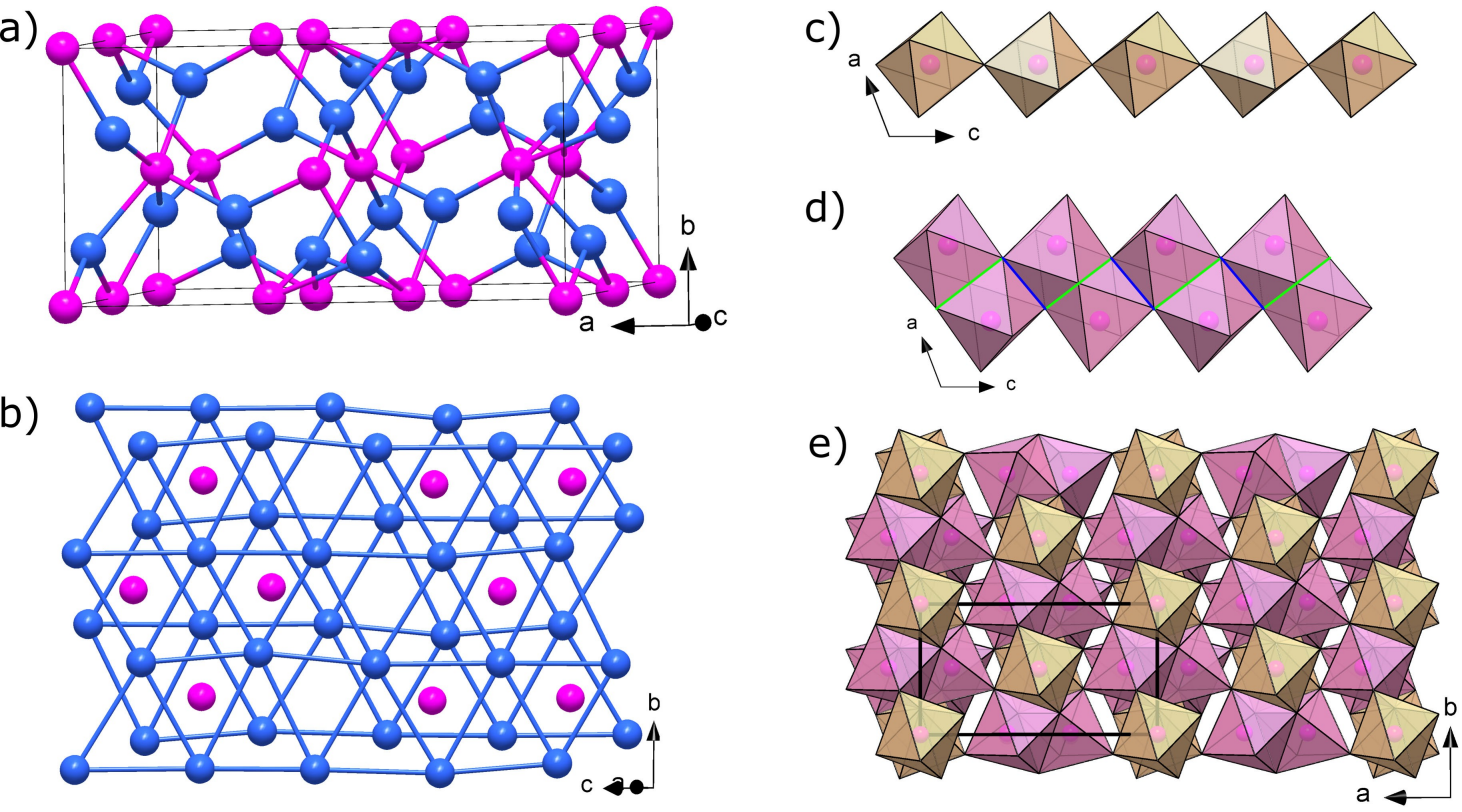
Structure of δ‐P_3_N_5_ at 72 GPa. a) The unit cell. b) The distorted hcp arrangement of the N atoms with P atoms filling 3/5 of the octahedral voids. c) Chains formed by the P2‐centered PN_6_ octahedra. d) Anatase (TiO_2_)‐like chains formed by the P1‐centered PN_6_ octahedra. The green and blue lines indicate face‐ and edge‐sharing PN_6_ units. e) Four unit cells with polyhedra drawn. The pink and blue spheres represent P and N atoms, respectively.

Here, we report the synthesis of three new phosphorus nitrides, namely α′‐P_3_N_5_, δ‐P_3_N_5_ and PN_2_, obtained through direct nitridation of elemental phosphorus. δ‐P_3_N_5_ and PN_2_ both feature the hitherto elusive PN_6_ octahedra. To accomplish this, three BX90‐type diamond anvil cells (DAC)^[29]^ were prepared to investigate the high‐pressure behavior of phosphorus–nitrogen samples up to 137 GPa. As described in detail in the Supporting Information, one DAC was loaded with red phosphorus and the two others with the black phosphorus allotrope. In either case the samples of phosphorus were loaded along with gaseous molecular nitrogen. At 72 GPa, laser‐heating a sample to temperatures above 2600 K resulted in the formation of a new solid, based on the appearance of new diffraction lines which could not be explained by the known phosphorus nitride γ‐P_3_N_5_,^[21]^ nitrogen (ϵ‐N_2_, ζ‐N_2_ or ι‐N_2_)[^30^, ^31^, ^32^] or phosphorus (phase III; Figure S1 in the Supporting Information).[^33^, ^34^] Single‐crystal X‐ray diffraction (SC‐XRD) of selected areas of the polycrystalline sample was performed and the collected data enabled a full structural solution (see Table S1 for the crystallographic details). The formed compound has a monoclinic unit cell (space group *C*2/*c*, no. 15) with lattice parameters *a*=8.418(5), *b*=4.325(6) and *c*=8.418(5) Å and *β*=110.96(7)°, *Z*=4, *V*=205.4(4) Å^3^. It is composed of five crystallographically unique atoms, P1 and P2, which are on the 8 f and 4a Wyckoff sites, and N1, N2 and N3 on the 4e, 8 f and 8 f Wyckoff positions, respectively. Having the P_3_N_5_ stoichiometry, this novel solid is hereafter named δ‐P_3_N_5_. δ‐P_3_N_5_ has the high‐temperature V_3_O_5_ structure type^[35]^ and was previously predicted from theoretical calculations.[^14^, ^22^] As shown in Figure 1, the two distinct P atoms are sixfold coordinated by nitrogen atoms, forming the sought after PN_6_ octahedra. The N1 and N2 atoms are both fourfold coordinated by phosphorus atoms and thus exhibit an ammonium type character, while the N3 atom is threefold coordinated by P atoms. The atoms’ environment can be expressed by the Niggli formula ${}_{\infty }^{3}\left[P_{3}^{\left[6\right]}N_{2}^{\left[3\right]}N_{3}^{\left[4\right]}\right]$ . δ‐P_3_N_5_ is the first phosphorus nitride featuring nitrogen atoms with ammonium type N^[4]^ character. However, the latter are known in γ‐Si_3_N_4_[^9^, ^36^] and in nitridosilicates.[^37^, ^38^] As expected, the average P−N^[4]^ distance (1.694(9) to 1.895(6) Å) is significantly longer than its P−N^[3]^ (1.600(6) to 1.644(5) Å) counterpart. The average of all P−N bonds in δ‐P_3_N_5_ is 1.712(6) Å in length, comparable to those found in γ‐P_3_N_5_ at 1 bar (average bond length of 1.69 Å).^[19]^ δ‐P_3_N_5_ was also synthesized at 118 GPa from laser‐heating red phosphorus and molecular nitrogen (Table S2).

The experimentally determined structural model of δ‐P_3_N_5_ was perfectly reproduced by Kohn‐Sham density functional theory (DFT) calculations (Table S1), and phonon calculations found the structure dynamically stable at 72 GPa (Figure S2). The computed electronic bands and projected electron density of states (p‐eDOS) of δ‐P_3_N_5_ show that it is a semiconductor with an indirect bandgap of 2.8 eV (Figure S3). This was calculated using the Perdew–Burke–Ernzerhof (PBE) approximation to exchange and correlation which likely underestimates the real band gap. The calculated electron localization function (ELF) (Figure S4) displays the polar‐covalent nature of the bonding between P and N. As expected, the analysis of ELF isosurfaces showed that both N^[4]^ centers do not display a lone electron pair, donated to the N−P dative bond (Figure S4). On the opposite, the N3 atom (N^[3]^ center), not expected to form a dative bond, shows a lone electron pair. Dative bonding can be considered as a natural response to a pressure increase, serving to extend both nitrogen's coordination and the compound's density.

The structural arrangement of δ‐P_3_N_5_ can be described as a distorted hexagonal closest packing (hcp) of N atoms with the 3/5 of octahedral voids filled with P atoms. This is similar to β‐BP_3_N_6_ which also features a nitrogen distorted hcp arrangement with P atoms in the octahedral voids, but additionally with B atoms in the tetrahedral cavities.^[27]^ The structure of δ‐P_3_N_5_ can also be broken down into two interconnected chainlike structural elements running along the [001] direction, as illustrated in Figure 1, with P atoms on *y*≈0 and *y*≈1/2. The first chain is composed of “double octahedra”, that is, two face‐sharing octahedra with the P1 atoms at their centers (P1−P1 distance of 2.407(5) Å at 72 GPa) that are linked together by their edges to form a chain. Exhibiting face‐sharing octahedra is an indication of the (polar) covalent bonding in δ‐P_3_N_5_; as known from Pauling's third rule,^[39]^ this configuration is very unfavorable for cations in ionic structures. The other type of chain, comprised of P2‐centered PN_6_ units, is composed of single octahedra linked by corner sharing. The two sets of chains are further crosslinked by edge‐ and corner‐sharing PN_6_ units, and alternate in both [100] and [010] directions.

Raman measurements on δ‐P_3_N_5_ were performed at 82 GPa and vibrations at frequencies of 416, 626, 673 and 838 cm^−1^ were detected. As shown in Figure S5, these modes match very well with those previously observed from an unidentified reaction product from a γ‐P_3_N_5_ sample laser‐heated between 67 and 70 GPa, thereby strongly suggesting it to be δ‐P_3_N_5_ as well.^[21]^

**Figure 2:**
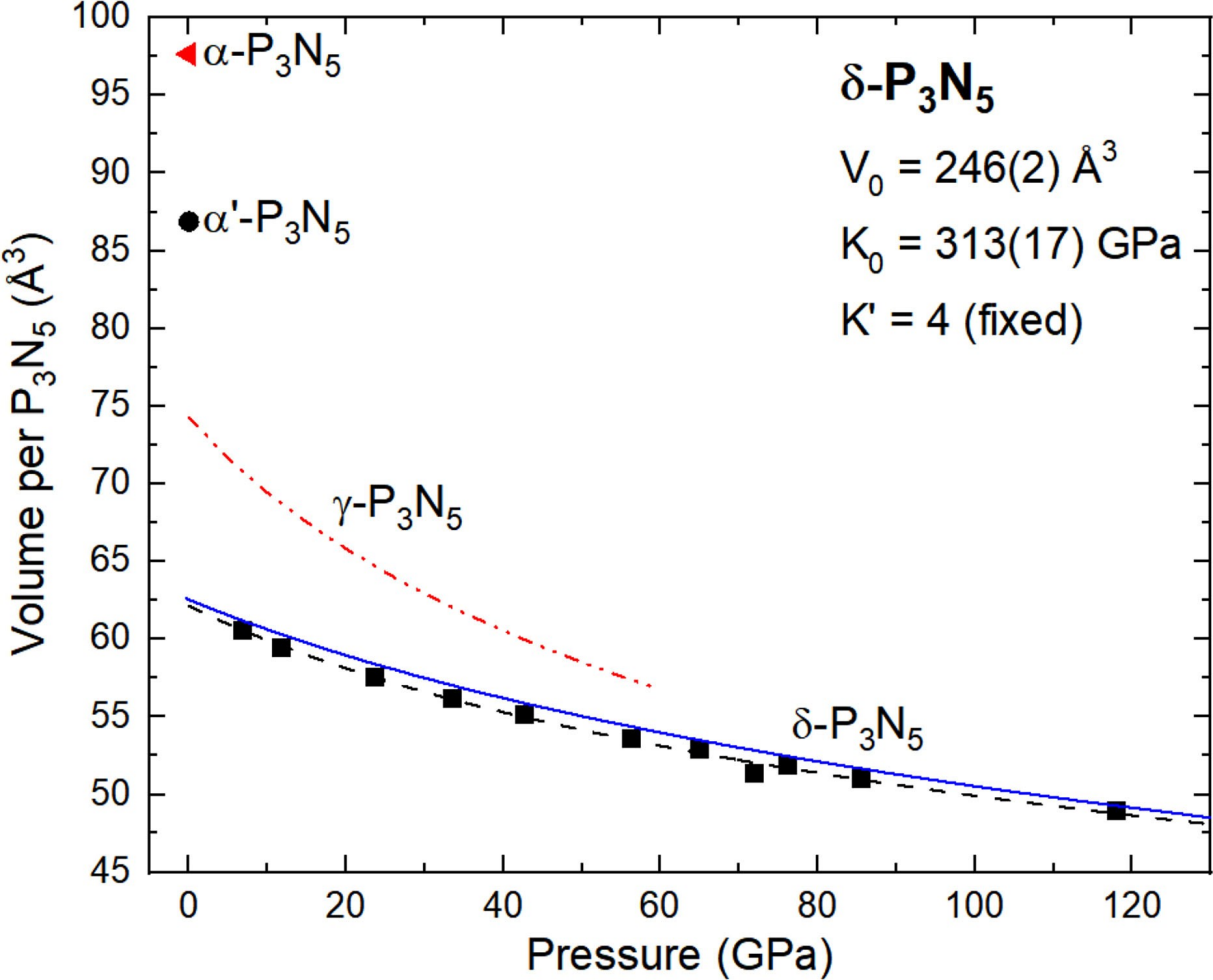
Volume per P_3_N_5_ unit with respect to pressure for all known polymorphs of P_3_N_5_. The black squares and circle represent experimental datapoints on δ‐P_3_N_5_ and α′‐P_3_N_5_ obtained in our experiments. The black dashed line is a fit of the δ‐P_3_N_5_ experimental data with a second order Birch–Murnaghan equation of state, with parameters of V_0_=245.7(6) Å^3^, K_0_=322(6) GPa and K′ fixed to a value of 4. The full blue line represents the calculated equation of state for δ‐P_3_N_5_. The red dot‐dashed line and the red triangle represent the literature experimental data on γ‐P_3_N_5_ ^[21]^ and α‐P_3_N_5_,^[18]^ respectively. β‐P_3_N_5_ is not included as its exact structure is not known, although it is likely to be a stacking variant of α‐P_3_N_5_.^[18]^

δ‐P_3_N_5_ produced at 72 GPa was characterized by SC‐XRD during its decompression. The full crystallographic data at 118 GPa is shown in Table S2, while the decompression data, with the unit cell parameters of δ‐P_3_N_5_ extracted, are shown in Figure 2 as well as in Table S4. The δ‐P_3_N_5_ polymorph was detected down to a minimum pressure of 7 GPa, and experimentally determined to have a bulk modulus of *K*_0_=322(6) GPa (V_0_=245.7(6) Å^3^, *K*′=4 (fixed); outlier point at 72 GPa not included in the fit). The incompressibility value, being above 300 GPa, qualifies δ‐P_3_N_5_ as an ultra‐incompressible solid similar to spinel‐type BeP_2_N_4_.^[28]^ Its incompressibility is vastly greater than that of the lower pressure polymorph γ‐P_3_N_5_, determined to have a *K*_0_=130.27(43) GPa (*K*′=4 (fixed)). This difference can easily be explained by the polymorphs’ respective crystal chemistry, that is, γ‐P_3_N_5_ being composed of a mixture of PN_4_ and PN_5_ units while δ‐P_3_N_5_ is exclusively made up of PN_6_ octahedra, even including face‐sharing.

Subsequently, the DAC containing δ‐P_3_N_5_ was fully opened, thereby releasing nitrogen gas and exposing the sample to air. Despite a thorough X‐ray diffraction mapping of the sample chamber, δ‐P_3_N_5_ could no longer be observed. However, diffraction lines from yet another novel solid were identified, and its crystal structure was solved from the collected single‐crystal data. This revealed the formation of a fifth P_3_N_5_ polymorph, hereafter named α′‐P_3_N_5_, which is very likely to be the phase transformation product of δ‐P_3_N_5_ at pressures below 7 GPa. Similar phase transformations of high‐pressure phases upon full pressure release have been observed in a number of systems, including BeN_4_ ^[40]^ and Mg_2_N_4_.^[41]^ The formation of α′‐P_3_N_5_ further emphasizes that compounds formed at high pressures can serve as precursors to explore a phase space otherwise inaccessible.

**Figure 3:**
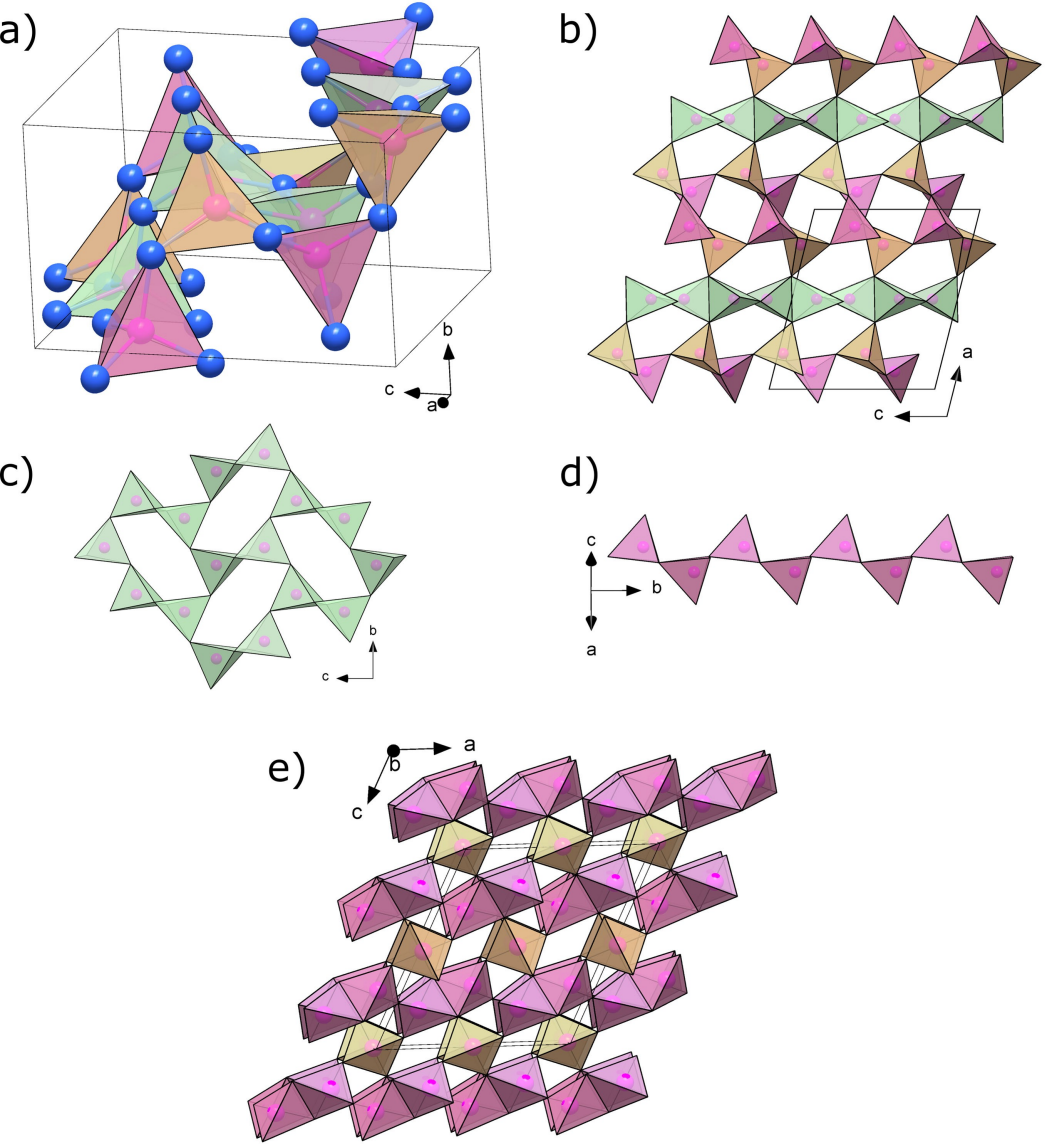
Structure of α′‐P_3_N_5_ at 1 bar. Blue and pink spheres represent N and P atoms, respectively. The light pink, light orange and light green PN_4_ tetrahedra are centered by P1, P2 and P3, respectively. a) Unit cell. b) Multiple unit cells viewed along the b‐axis, showing the stacking of the different tetrahedra along the a‐axis. c) The arrangement of the light green (P3 centered) tetrahedra in the bc‐plane. Each of four distorted rings shown is composed of four corner‐sharing and two edge‐sharing PN_4_. Such an arrangement of tetrahedra has previously been observed in HP−NiB_2_O_4_.^[42]^ d) Single zweier chain of light pink tetrahedra (P1 centered) running along the b‐axis. e) For comparison, the structure of α‐P_3_N_5_ at 1 bar viewed along the [010] direction.^[18]^ PN_4_ tetrahedra forming zweier single chains are drawn in light pink, and are alternately corner‐ and edge‐sharing. The chains are connected together by additional PN_4_ tetrahedra, drawn in light orange.

At ambient conditions, α′‐P_3_N_5_ was found to have a monoclinic unit cell (space group *P*2_1_/*c*, no. 14) with lattice parameters *a*=9.2557(6), *b*=4.6892(3), *c*=8.2674(6) Å and *β*=104.160(6)°, *Z*=4, *V*=347.92(5) Å^3^. All eight crystallographically distinct atoms, three P and five N, are occupying 4e Wyckoff sites. The full crystallographic data is available in Table S3 and reciprocal space unwarps shown in Figure S6. As shown in Figure 3, the structure is composed of crosslinked PN_4_ tetrahedra and has the Niggli formula ${}_{\infty }^{3}\left[P_{3}^{\left[4\right]}N_{3}^{\left[2\right]}N_{2}^{\left[3\right]}\right]$ – the same as the known ambient condition polymorph α‐P_3_N_5_ (Figure 3),^[18]^ and similar to β‐Si_3_N_4_ ${}_{\infty }^{3}\left[Si_{3}^{\left[4\right]}N_{4}^{\left[3\right]}\right]$ . When viewed along the *b*‐axis, it can be understood as two types of PN_4_ layers (light green and light pink tetrahedra) joined together by connecting corner‐sharing PN_4_ units (light orange tetrahedra). The light green tetrahedra, centered by P3, are forming distorted rings composed of six tetrahedra in the *bc*‐plane, four corner‐sharing and two edge‐sharing, an arrangement of tetrahedra which had previously been observed in HP−NiB_2_O_4_.^[42]^ The light pink tetrahedra (P1 atom at their center) are producing a corner‐sharing single zweier chain (according to Liebau's^[43]^ nomenclature) running along the *b*‐axis. While (non‐condensed) zweier chains also occur in Mg_2_PN_3_ and Ca_2_PN_3_,[^44^, ^45^] the crystal structure of α′‐P_3_N_5_ is, to the best of our knowledge, a new structure type. At 1 bar, the P−N bond lengths vary between 1.51(1) Å and 1.78(1) Å, similar to those in α‐P_3_N_5_ (1.51 to 1.74 Å).^[18]^ Still, α′‐P_3_N_5_ is found to be 12.4 % denser than α‐P_3_N_5_, as it can be deducted from Figure 2. This can be explained by the tighter packing of phosphorus atoms in α′‐P_3_N_5_, with an average P−P distance of 2.900(5) Å compared to 2.98 Å in α‐P_3_N_5_.^[18]^

DFT calculations on α′‐P_3_N_5_ agree with the experimental structural model (Table S3) and demonstrate its dynamical stability (Figure S7). Akin to δ‐P_3_N_5_, the P−N bonds in α′‐P_3_N_5_ are seen from ELF calculations to be polar covalent (Figure S8). α′‐P_3_N_5_ is found to be a wide band gap semiconductor, having a calculated direct band gap of 3.45 eV (using PBE, Figure S9), which is significantly lower than the calculated indirect bandgap of 5.21 eV for α‐P_3_N_5_.^[7]^ Despite α′‐P_3_N_5_ and α‐P_3_N_5_ featuring many similarities regarding their crystal chemistry, as exemplified by their identical Niggli formula, the much smaller band gap of α′‐P_3_N_5_ can be hypothesized to be a consequence of its higher density, leading to a more thorough overlap of the electronic orbitals. The bulk modulus of α′‐P_3_N_5_ is calculated to be *K*_0_=95.4 GPa (*K*′=3.89), which is in line with that of α‐P_3_N_5_ (*K*_0_=87 GPa (*K*′=2.0) or 99 GPa (*K*′=1.9), depending on the calculations.^[23]^

**Figure 4:**
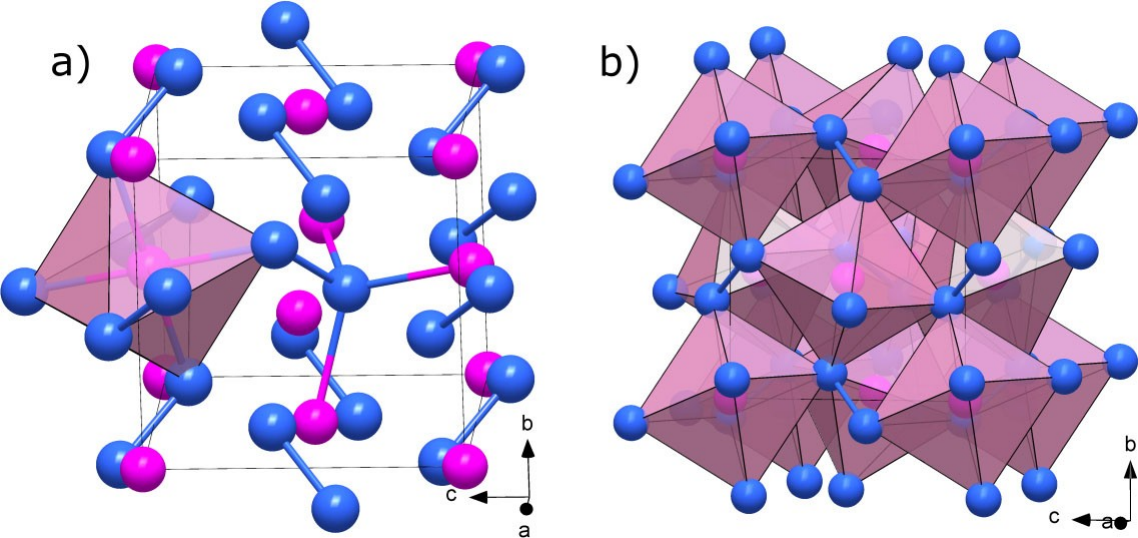
Crystal structure of PN_2_ at 134 GPa, where the blue and pink spheres represent nitrogen and phosphorus atoms, respectively. a) Unit cell with a single PN_6_ octahedra and the four bonds of the N^[4]^ center drawn. b) Unit cell with all PN_6_ octahedra shown.

Further experiments on the phosphorus‐nitrogen system were also conducted above 120 GPa. Sample laser‐heating to temperatures above 2000 K at 134 GPa resulted in the formation of yet another binary phosphorus nitride with the unprecedented PN_2_ stoichiometry. Its crystal structure was solved from SC‐XRD data at both 134 GPa as well as at 137 GPa after further compression (Tables S5 and S6; see Figure S10 for reciprocal space unwarps). PN_2_ has a simple pyrite‐type structure (cubic, space group *Pa*‐3, no. 205)‐akin to group 14 element nitrides SiN_2_, GeN_2_ and SnN_2_ ^[15]^ as well as the platinum group metal nitrides PtN_2_ and PdN_2_[^46^, ^47^, ^48^, ^49^] and has a unit cell parameter *a*=4.0127(14) Å with *Z*=4 and *V*=64.61(4) Å^3^) at 134 GPa. As shown in Figure 4, it is composed of two chemically and crystallographically distinct atoms, P1 and N1, respectively on Wyckoff positions 4a and 8c, respectively. PN_2_ is a 3D polymeric compound that can be expressed by the Niggli formula ${}_{\infty }^{3}\left[P_{1}^{\left[6\right]}N_{2}^{\left[4\right]}\right]$ and is composed of corner‐sharing PN_6_ octahedra crosslinked together through nitrogen dimers. Each nitrogen atom is therefore forming three P−N bonds (1.699(3) Å, 134 GPa) and a single N−N bond (1.418(6) Å, 134 GPa). The P−N bond length is marginally shorter than the average P−N contacts in δ‐P_3_N_5_ at 72 GPa (1.712(6) Å), whereas the length of the nitrogen dimer strongly suggests a single‐bond, that is, a pernitride unit ([N_2_]^4−^).^[50]^ From the Niggli formula of the PN_2_ compound, the N^[4]^ center is also likely to feature a dative bond with phosphorus.

Although the electron distribution in P_3_N_5_ polymorphs is well‐determined in the purely ionic approximation (i. e., P^V+^_3_N^III−^_5_),^[20]^ it is not as trivial in PN_2_. The most likely configuration is [P^5+^][N_2_]^4−^⋅e^−^, in analogy with metallic binary subnitrides such as Ba_2_N ([Ba^2+^]_2_[N^3−^]⋅e^−^).^[51]^ This interpretation is strengthened by the bond length of the N_2_ dimers, fitting that of a pernitride. The metallic character of PN_2_ suggested from this analysis is compatible with the lack of a measurable Raman signal from the sample.

In order to analyze the stability of PN_2_, variable cell structural relaxations at 137 GPa were performed. If atomic positions were allowed to freely relax, only a small difference in lattice constants of ∼2 % was found, but the distance between nitrogen atoms increased to ∼1.9 Å compared to the experimentally‐obtained ∼1.4 Å (see Supporting Information). However, the relaxed structure features negative modes in its phonon dispersion relations calculated at *T*=0 K in the harmonic approximation (Figure S11). In order to investigate the possibility of a temperature induced dynamical stabilization of PN_2_, ab‐initio as well as classical molecular dynamics (MD) simulations based on a machine learned inter‐atomic potential were performed (see the Supporting Information). In both cases, no sign of a dynamical instability of PN_2_ during the MD simulations at 300 K was observed. However, the N−N distances were strongly modified, independent of the starting configuration (all 1.4 or all 1.9 Å), and a mixture of ∼37.5 % ∼1.4 Å (i. e., single‐bonded N−N) and ∼62.5 % ∼2 Å N−N distances (i. e., no N−N bonds) appeared (Figure S12 and Figure S13). Increasing the temperature to the experimental synthesis temperature of 2500 K led to an increased number of ∼1.4 Å N−N distances, that is, ∼47 % (Figure S14). This mixed bonding arrangement is, independent of temperature, overall stable over the full ∼10 ps length of the ab‐initio MDs (92 atoms) and also stable up to 24 ns in the classical MDs (192 atoms), but N−N distances are found to switch between the two states dynamically.

To test whether this disordered theoretical model was a better fit to the experimental single‐crystal XRD data, the data was additionally analyzed with the N1 position split into two (N1 and N1′) and their occupancy (sum constrained to 1) as well as coordinates refined independently. An occupancy of 92 % for the N1 atom and 8 % for the N1′ atom was obtained, with N1−N1 and N1′−N1′ distances of 1.3802(3) and 1.9820(3) Å, respectively. This disordered model has equal or slightly higher reliability factors (*R* factors) than the original model, substantiating that the calculations capture the overall nature of the structure, showing a disorder in the N−N distances.

Additional theoretical calculations were performed to analyze the electronic configuration, including the assumed metallic character of the PN_2_ solid. Calculations were performed for unit cell models without, with 25 % and with 100 % N−N single bonds. As seen in Figure S15, all models were found to have a non‐zero DOS at the Fermi energy, demonstrating the metallicity of the PN_2_ compound, in agreement with the electron distribution inferred from the compound's crystal chemistry. Computed ELFs, for example with 25 % N−N bonds shown in Figure S16, nonetheless display the strong covalent nature of the P−N interaction, and the lack of lone electron pairs on the nitrogen atoms confirms the expected dative bonding between nitrogen and phosphorus.

Attempts to decompress PN_2_ resulted in an additional datapoint at 109 GPa, below which the compound could no longer be observed. According to our calculations, PN_2_ is predicted to have a bulk modulus of *K*_0_=339.0 GPa (*V*_0_=86.3 Å^3^ and *K*′=4.21), therefore being even more incompressible than δ‐P_3_N_5_ and qualifying it as an ultra‐incompressible solid. This can be explained by the known incompressibility of compounds containing pernitride units, combined with the higher overall nitrogen coordination of PN_2_ (i. e., only N^[4]^ centers for PN_2_ vs. a 3 : 2 mixture of N^[4]^/N^[3]^ for δ‐P_3_N_5_).

The synthesis of δ‐P_3_N_5_ and PN_2_, both containing PN_6_ units, brings phosphorus nitrides‐and pnictogen nitrides as a whole‐in line with their periodic table neighbor silicon, germanium and tin nitrides (group 14 element nitrides) as well as sulfur nitrides (chalcogen nitrides), all of which feature XN_6_ octahedra (X=Si, Ge, Sn and S).[^15^, ^16^] Whereas the Si‐, Ge‐ and Sn‐pernitrides, SiN_2_, GeN_2_, SnN_2_, were all produced around 60 GPa,^[15]^ the formation of the SN_6_ units in SN_2_ required a significantly higher pressure of 120 GPa.^[16]^ In the case of PN_6_, an intermediate pressure of 72 GPa was found sufficient. These formation pressures qualitatively match with the covalent radius (and electronegativity) of the non‐nitrogen atom, with the larger elements (Sn, Ge, Si) requiring less pressure than the smaller ones (S)‐according to the pressure homologue rule. It is interesting to note that pernitrides with the same formula type SiN_2_, GeN_2_, SnN_2_, PN_2_ and SN_2_ are possible, and all but SN_2_ adopt the pyrite‐type structure with N^[4]^ centers.^[15]^ SN_2_ has the CaCl_2_‐type structure with only N^[3]^ centers,^[16]^ and therefore a further increase in nitrogen coordination is expected to be achieved at higher pressures.

## Conclusions

**Figure 5:**
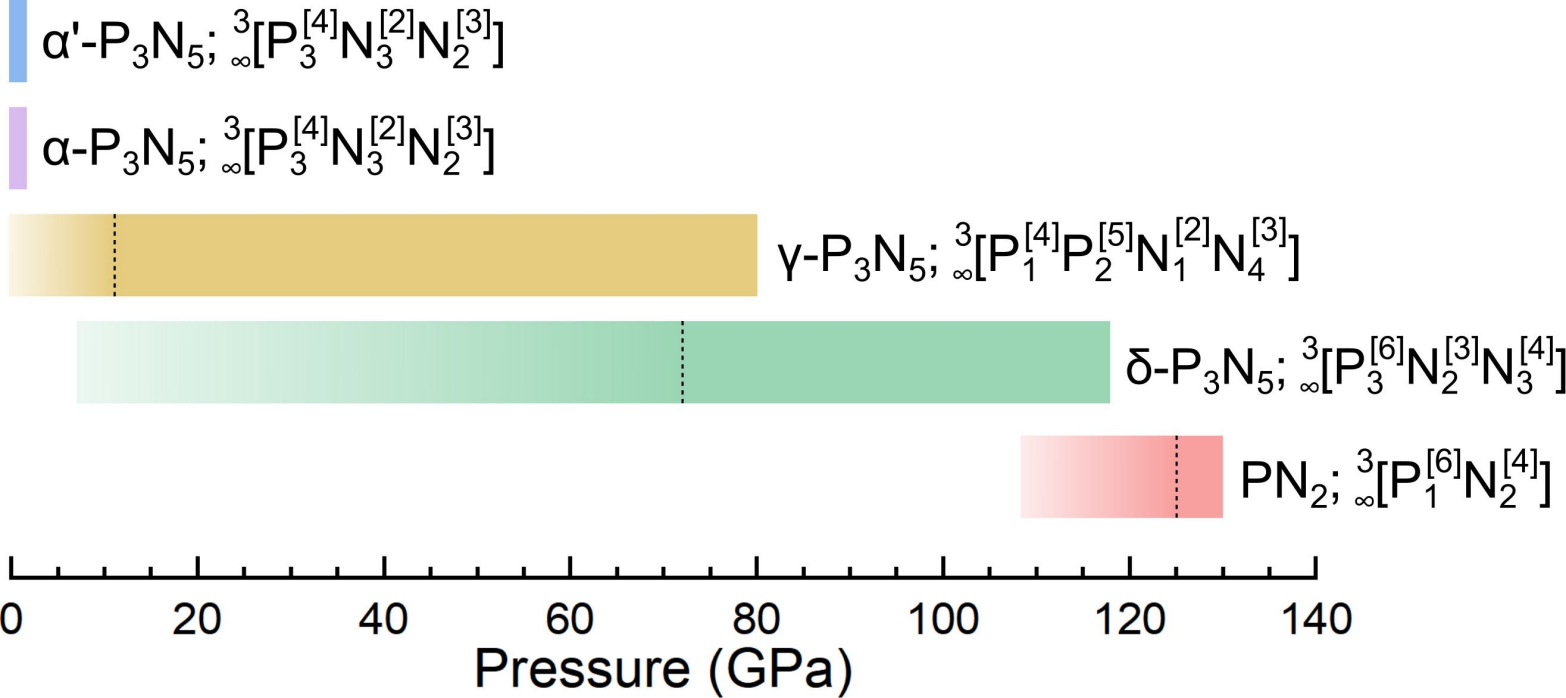
Schematic representation of the stability domains of the phosphorus–nitrogen system at high pressures. The colored horizontal lines show the pressure range at which a given phase was observed. The black vertical dashed line marks the lowest pressure at which a given compound was synthesized after laser‐heating. Each compound's Niggli representation is provided alongside its name. α′‐ and α‐P_3_N_5_ were observed solely at 1 bar.

The results presented in this study, summarized in Figure 5, extend our understanding of the phosphorus–nitrogen system to a pressure of 137 GPa. Three previously unidentified nitride phases have been discovered, including two polymorphs of P_3_N_5_ (α′‐P_3_N_5_ and δ‐P_3_N_5_) as well as the pernitride PN_2_. Most importantly, δ‐P_3_N_5_ and PN_2_ both feature the PN_6_ unit, long sought‐after in binary phosphorus nitrides, and bridge an important gap between group 14 element nitrides and chalcogen nitrides. Dative bonding is suggested by DFT calculations to enable the N^[4]^ centers in δ‐P_3_N_5_ and PN_2_‐a first observation in phosphorus nitrides. The presence of both the PN_6_ octahedra and the N^[4]^ centers in δ‐P_3_N_5_ and PN_2_ provides a clear explanation for their very high incompressibilities of 322(6) and 339.0 GPa, respectively. δ‐P_3_N_5_ is found to be non‐recoverable under ambient conditions as it transforms into α′‐P_3_N_5_ below 7 GPa. Although α′‐P_3_N_5_ has the same PN_4_ constituting units as α‐P_3_N_5_, as well as the same atomic coordination, it is of significantly higher density (12.4 %). In the same way that α‐P_3_N_5_ was considered for technological applications,[^5^, ^52^] further studies on α′‐P_3_N_5_ are necessary to assess its potential range of applicability. This investigation should stimulate further high‐pressure, high‐temperature experiments of non‐metal nitrides‐largely neglected compared to metal nitrides‐in order to gain a deeper understanding of their fundamental chemistry, which will contribute to the discovery of recoverable materials with uses in everyday life.

## Experimental Section

**Experimental methods**: Three BX90‐type screw‐driven diamond anvil cell (DAC)^[29]^ were prepared. One was equipped with 120 μm culet diamond anvils (120‐DAC) and two others with 80 μm culet diamond anvils (80‐DAC). Rhenium gaskets with an initial thickness of 200 μm were indented down to ∼12–18 μm and sample cavities of about 40 to 60 μm in diameter were laser‐drilled at the center of the indentations. One 80‐DAC was loaded with red phosphorus (99.999 %, Puratronics) while the two other DACs were loaded with black phosphorus. The procedure for producing black phosphorus is described in further detail below.^[53]^ In both cases, the phosphorus piece was loaded alongside molecular nitrogen gas (∼1200 bars), acting as a reagent and a pressure transmitting medium. The in‐situ pressure was measured using the first‐order Raman mode of the stressed diamond anvils^[54]^ and verified through X‐ray diffraction measurements by comparing to the pressure obtained from the calibrated equation of state of the rhenium gasket. Double‐sided sample laser‐heating was performed at our home laboratory at the Bayerisches Geoinstitut (BGI)^[55]^ as well as at the GSECARS (APS) and P02.2 (PETRA III) beamlines with the phosphorus precursor employed as the laser absorber. Temperatures were measured with an accuracy of ±200 K, using the thermoemission produced by the laser‐heated samples.^[55]^

Synchrotron X‐ray diffraction measurements of the compressed samples were performed at ID11 (*λ*=0.2852 Å) and ID15 (*λ*=0.41015 Å) of the ESRF‐EBS, at the P02.2 beamline (0.2910 Å) of DESY as well as at the GSECARS beamline (*λ*=0.29521 Å) of the APS. In order to determine the position of the polycrystalline sample on which the single‐crystal X‐ray diffraction (SC‐XRD) acquisition is obtained, a full X‐ray diffraction mapping of the pressure chamber was achieved. The sample position displaying the most and the strongest single‐crystal reflections belonging to the phase of interest was chosen for the collection of single‐crystal data, collected in step‐scans of 0.5° from −38° to +38°. The CrysAlisPro software^[56]^ was used for the single crystal data analysis. The analysis procedure includes the peak search, the removal of the diamond anvils’ and other “parasitic” signal contributions, finding reflections belonging to a unique single crystal, the unit cell determination, and the data integration. The crystal structures were then solved and refined using the OLEX2 and JANA2006 software.[^57^, ^58^] The SC‐XRD data acquisition and analysis procedure was previously demonstrated and successfully employed.[^16^, ^59^, ^60^, ^61^] The full details of the method can be found elsewhere.^[62]^ Powder X‐ray diffraction measurements were also performed to verify the sample‘s chemical homogeneity. The powder X‐ray data was integrated with the Dioptas software.^[63]^

Confocal Raman spectroscopy measurements were performed with a LabRam spectrometer equipped with a ×50 Olympus objective. Sample excitation was accomplished using a continuous He−Ne laser (632.8 nm line) with a focused laser spot of about 2 μm in diameter. The Stokes Raman signal was collected in a backscattering geometry by a CCD coupled to an 1800 l/mm grating, allowing a spectral resolution of approximately 2 cm^‐1^. A laser power of about 30 mW incident on the DAC was employed.

Black phosphorus was synthesized out of red phosphorus using the 5000 tons uniaxial split sphere apparatus (Voggenreiter Zwick 5000^[64]^) at the BGI, under conditions of 2 GPa and 600 °C maintained for 2 h. Lumps of red phosphorus (99.999 %, Puratronics) were crushed in an agate mortar and subsequently loaded into a hexagonal boron nitride capsule to avoid any chemical reaction or oxidation during the synthesis. This capsule was then inserted into a 25/15 (octahedral edge length/anvil truncation edge length, in millimeter) BGI standard multi‐anvil assembly equipped with a graphite heater. Temperature was monitored using a D‐type thermocouple and kept constant during the time of the synthesis

**Density functional theory calculations**: Kohn‐Sham density functional theory based structural relaxations and electronic structure calculations were performed with the QUANTUM ESPRESSO package[^65^, ^66^, ^67^] using the projector augmented wave approach.^[68]^ We used the generalized gradient approximation by Perdew‐Burke‐Ernzerhof (PBE) for exchange and correlation, with the corresponding potential files: for P the 2p electrons and lower and for N the 1 s electrons are treated as scalar‐relativistic core states. We include van der Waals corrections following the approach by Grimme et al. as implemented in Quantum Espresso.^[69]^ Convergence tests with a threshold of 1 meV per atom in energy and 1 meV/Å per atom for forces led to a Monkhorst‐Pack^[70]^ k‐point grid of 8x16x8 for both α′‐P_3_N_5_ and δ‐P_3_N_5_ as well as PN_2_ with a cutoff for the wave‐function expansion of 100 Ry for all phases. Phonon calculations were performed with PHONOPY^[71]^ in 2x3x2 supercells for α′‐ and δ‐P_3_N_5_ and 3x3x3 supercells for PN_2_ with respectively adjusted k‐points.

We performed variable cell relaxations (lattice parameters and atomic positions) on all experimental structures to optimize the atomic coordinates and the cell vectors until the total forces were smaller than 10^−4^ eV Å^−1^ per atom and the deviation from the experimental pressure was below 0.1 GPa

Furthermore, we calculated the equation of states (EOS) of both phosphorus nitrides by performing variable cell relaxations to respective target pressures until forces are <10^−3^ eV Å^−1^ and until the pressure is matched within 0.1 GPa. A third order Birch Murnagham (BM3) EOS was fitted to the calculated energy versus volume points. We obtained the following:

α′‐P_3_N_5_: *K*_0_=95.4 GPa,  *K*'=3.89, *V*_0_ =356.0 Å^3^
δ‐P_3_N_5_: *K*_0_=299.0 GPa,  *K*'=4.21, *V*_0_=125.1 Å^3^

We benchmarked the target pressure in the relaxations against the pressure obtained from the BM3 fit to ensure convergence. The EOS are in good agreement with experimental data obtained during compression and decompression (Figure 2).

In order to analyze the effect of temperature on the stability of the PN_2_ phase and the N−N distances connecting the PN_6_ octahedra, we ran ab‐initio molecular dynamics (MD) simulations with Quantum Espresso using 2x2x2 supercells and 2x2x2 k‐points and a timestep of 0.9697 fs. Simulations ran for 9.289 ps (300 K) and 9.869 ps (2500 K). We calculate the interatomic distance for each N−N pair as well as the percentage of short (<1.6 Å) and long (>1.6 Å) N−N distances in each timestep and obtain Figures S9 and S10. We find a stabilization of configurations with 37.5 % short distances at 300 K and 47 % short distances at 2500 K after ∼3 and ∼6 ps, respectively. To obtain a better understanding of the influence of cell size and simulation duration, we trained a moment tensor (mtp)^[72]^ machine learning interatomic potential using the MLIP code^[73]^ based on the structural relaxations and phonon calculations performed for PN_2_, which was refined using an active learning process.^[73]^ We trained the mtp potentials using ab‐initio calculated total energies, interatomic forces and stresses of supercells with 192 atoms. For the ab‐initio calculations in the active learning process, we used the Vienna ab‐initio simulation package (VASP)[^74^, ^75^, ^76^, ^77^] with the PBE generalized gradient approximation, 600 eV cutoff energy for the basis set and 4x4x4 sampling of the Brillouin zone. The final interatomic potential based on a 20 g.mtp^[72]^ was trained from 300 up to 2500 K and 110 GPa up to 150 GPa. We evaluate the effect of enhanced cell size (up to 3000 atoms) and increased simulation time (up to 24 ns) at various temperatures and pressures through trajectory calculations with Lammps^[78]^ using 1 fs timestep, calculating the time evolution of the radial distribution function (RDF). We find a very good agreement between the ab‐initio MD and classical MD simulations: independently of the chosen cell size, simulation time and initial atomic arrangements the system evolves in a structural state with N−N distances of ∼1.4 and ∼2 Å (cf. Figure S14).

Furthermore, we cannot find any influence greater than 2 % on the N−N distances performing calculations using the Heyd‐Scuseria‐Ernzerhof hybrid functional^[79]^ with the standard screening parameter, with and without van der Waals correction or spin polarization (magnetization along the *z*‐axis and noncollinear calculations with QE and VASP) starting from ferromagnetic and anti‐ferromagnetic spin arrangements that could account for the remaining differences in interatomic N−N distances.

## Data Availability

Deposition Numbers 2178817 (for δ‐P_3_N_5_ at 72 GPa), 2178818 (for δ‐P_3_N_5_ at 118 GPa), 2178819 (for α′‐P_3_N_5_ at 1 bar), 2178820 (for PN_2_ at 134 GPa), and 2178821 (for PN_2_ at 137 GPa) contain the supplementary crystallographic data for this paper. These data are provided free of charge by the joint Cambridge Crystallographic Data Centre and Fachinformationszentrum Karlsruhe Access Structures service. Other data that support the findings of this study are available from the corresponding author upon reasonable request.

## Conflict of interest

The authors declare no conflict of interest.

1

## Supporting information

As a service to our authors and readers, this journal provides supporting information supplied by the authors. Such materials are peer reviewed and may be re‐organized for online delivery, but are not copy‐edited or typeset. Technical support issues arising from supporting information (other than missing files) should be addressed to the authors.

Supporting Information

## Data Availability

The data that support the findings of this study are available from the corresponding author upon reasonable request.
