# Supplementary material for: Revealing Phosphorus Nitrides up to the Megabar Regime: Synthesis of α′‐P3N5, δ‐P3N5 and PN2
Source: Chemistry. 2022 Oct 17;28(62):e202201998. doi: 10.1002/chem.202201998 (PMC9827839; doi:10.1002/chem.202201998)
Supplement: Supplementary file 1 — Supporting Information [file CHEM-28-0-s001.pdf]

Chemistry–A European Journal

Supporting Information

Revealing Phosphorus Nitrides up to the Megabar Regime: Synthesis of α′-P_3N_5, δ-P_3N_5 and PN_2

Dominique Laniel,* Florian Trybel,* Adrien Néri, Yuqing Yin, Andrey Aslandukov, Timofey Fedotenko, Saiana Khandarkhaeva, Ferenc Tasnádi, Stella Chariton, Carlotta Giacobbe, Eleanor Lawrence Bright, Michael Hanfland, Vitali Prakapenka, Wolfgang Schnick, Igor A. Abrikosov, Leonid Dubrovinsky, and Natalia Dubrovinskaia

Table of Contents

Supporting Figures…………………………………………………………………………………… 2

Supporting Tables..……………………………………………………………………………...… 18

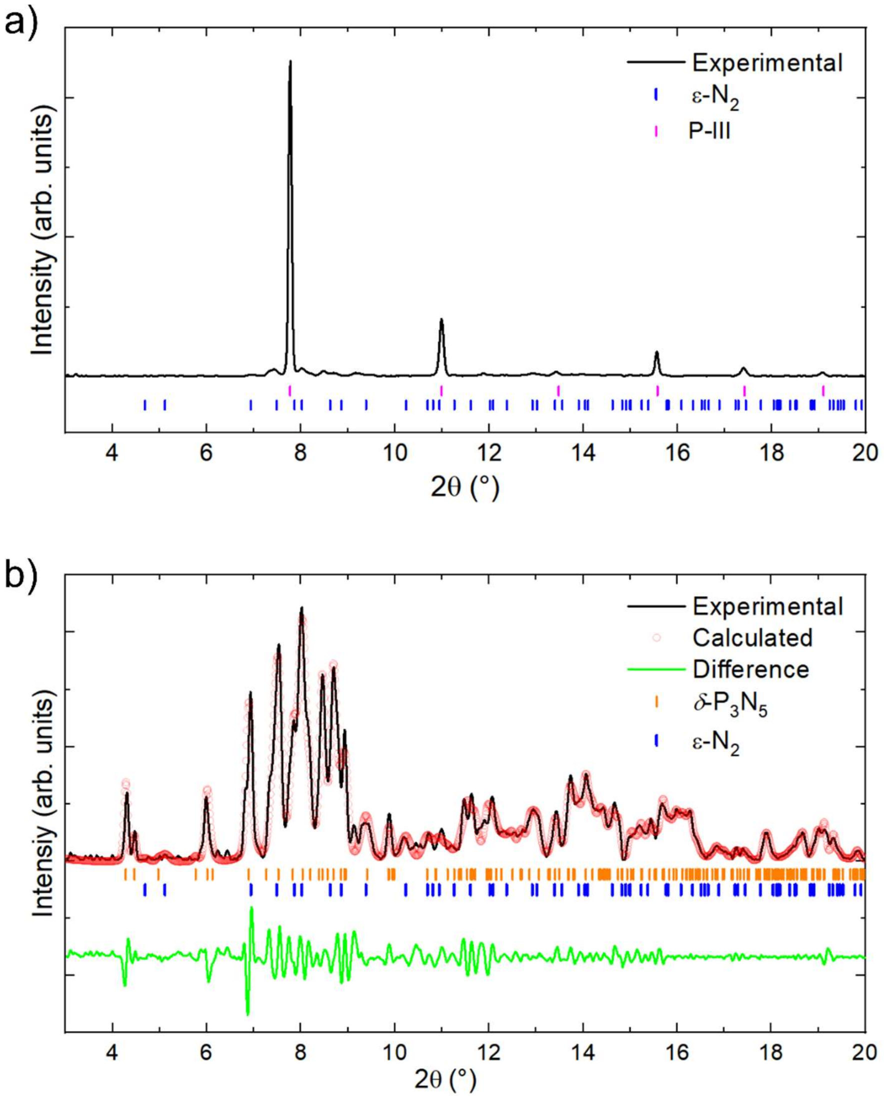

Figure S1: a) Integrated X-ray diffraction pattern collected at 72 GPa before laser-heating black phosphorus embedded in pure molecular nitrogen. b) Integrated X-ray diffraction pattern collected after laser-heating. New diffraction lines have appeared and can be indexed with the monoclinic (*C*2/*c* space group) unit cell of δ-P_3N_5. The integrated X-ray diffraction pattern is properly fitted with a Le Bail refinement of the unit cells of δ-P_3N_5 and ε-N_2. The corresponding patterns were collected with a wavelength of λ = 0.29521 Å.

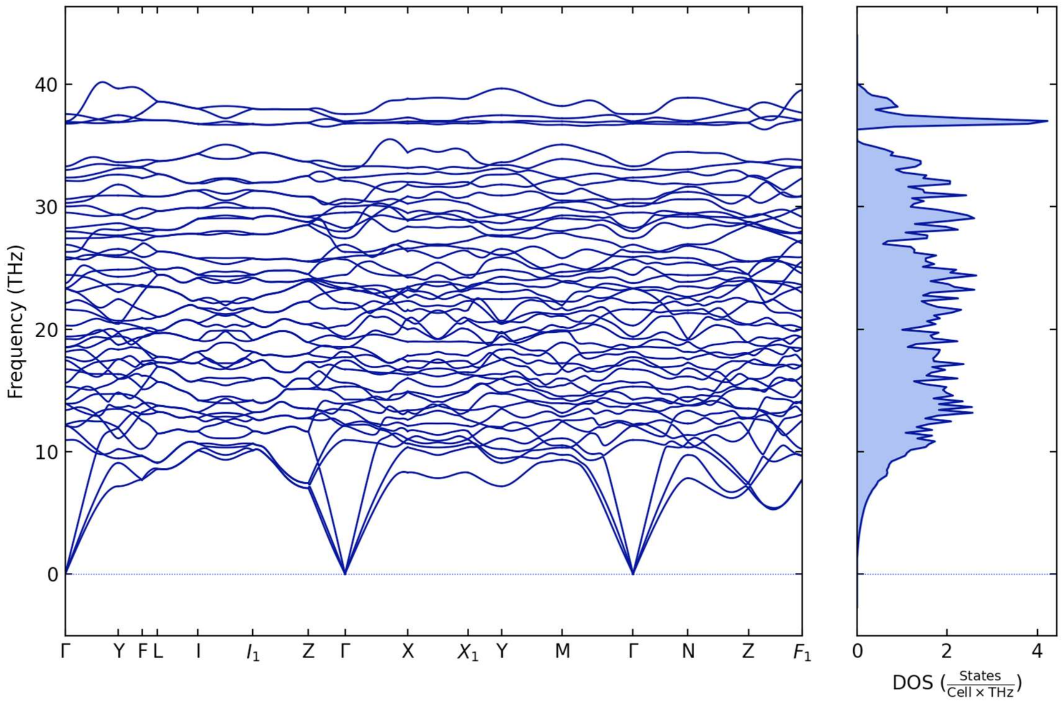

Figure S2: Calculated phonon density of state of δ-P_3N_5 at 72 GPa.

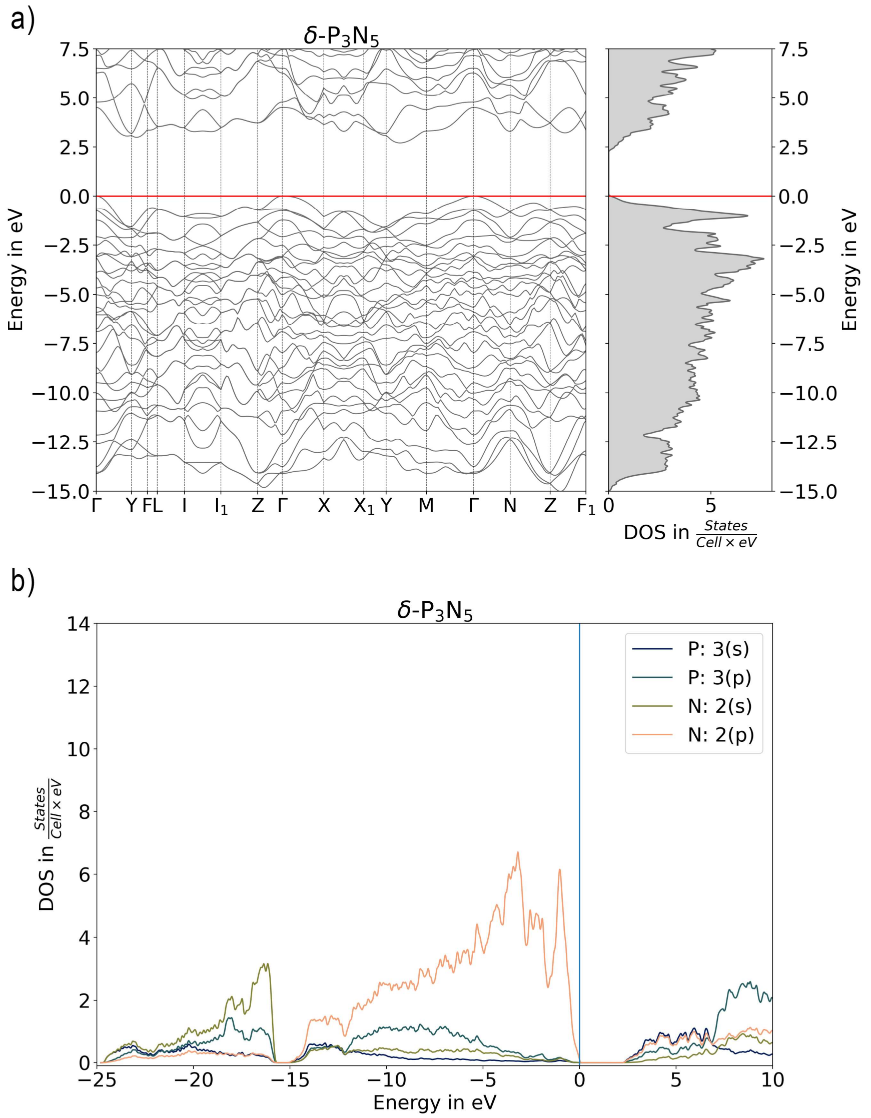

Figure S3: Electronic structure of δ-P_3N_5 at 72 GPa: a) electronic band structure and total electronic density of states and b) orbital projected electronic density of states.

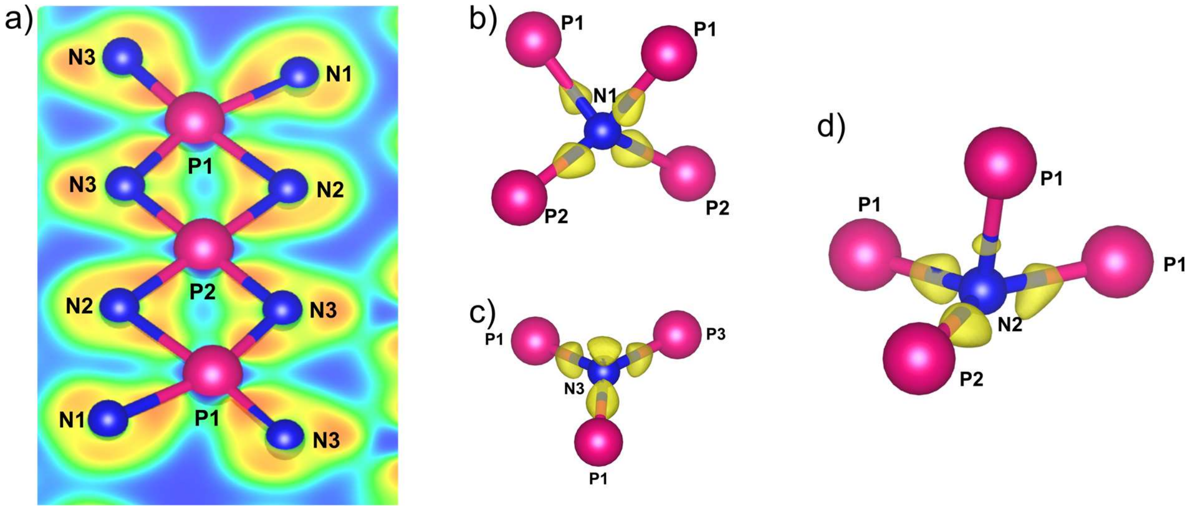

Figure S4: a) Electron localization function (ELF) slice δ-P_3N_5 at 72 GPa showing the polar covalent bonding between phosphorus and nitrogen atoms. b), c) and d) ELF isosurface at 0.85 e^-/Å^3 around the N1, N2 and N3 atoms. The N1 and N2 atoms do not have a lone electron pair as these nitrogen atoms are each making four bonds with phosphorus—supposedly through dative bonding. The N3 atom, forming only three P-N bonds, is seen to have a lone electron pair. The pink and blue spheres represent, respectively, phosphorus and nitrogen atoms.

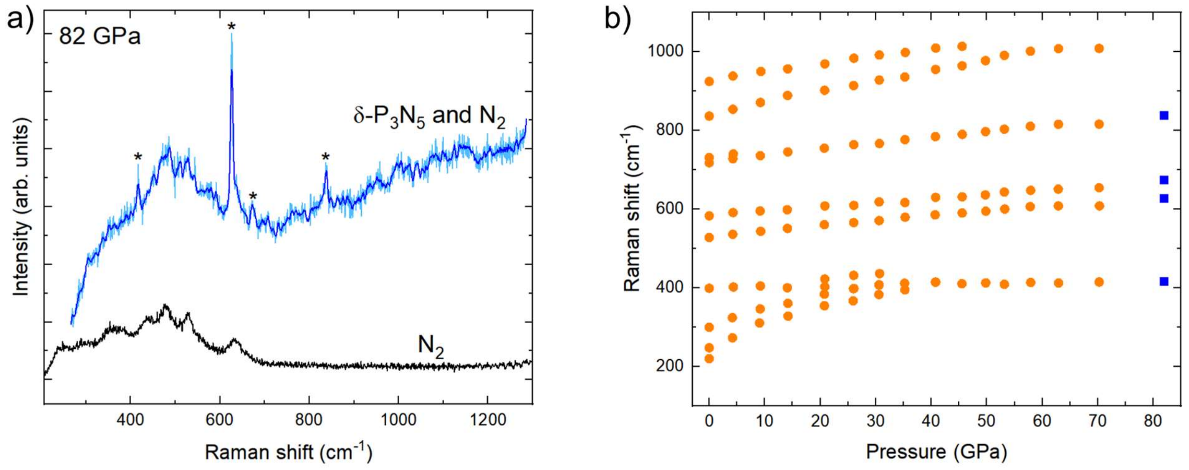

Figure S5: a) Raman spectra collected at 82 GPa on a P-N sample laser-heated at 72 GPa. The black spectrum was collected on pure molecular nitrogen while the light blue spectrum was collected on the produced δ-P_3N_5. The dark blue line is the smoothed experimental spectrum which helps guide the eye. The asterisks mark the peaks that were identified as belonging to δ-P_3N_5. b) Position of the experimentally-measured Raman modes of δ-P_3N_5 with pressure. The orange circles are datapoints from Niwa *et al* (2021)[30] and the blue squares those identified by asterisks in a).

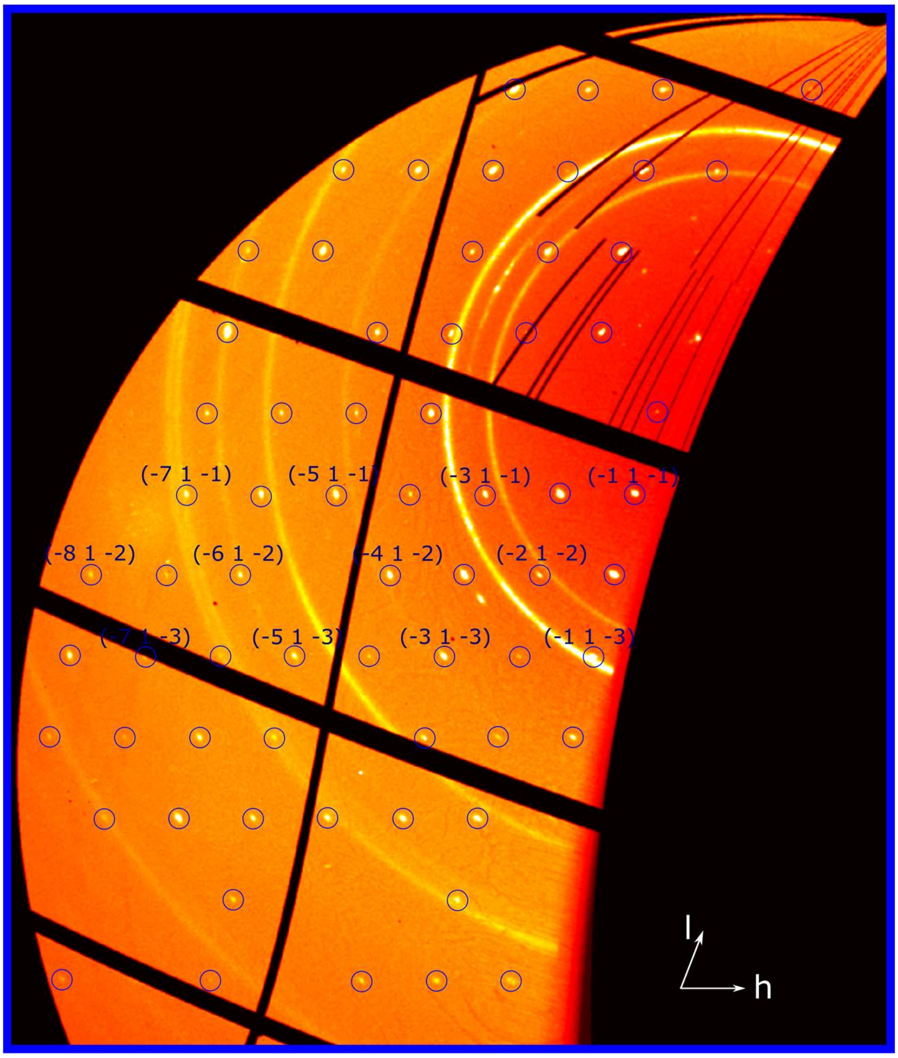

Figure S6: Slice of the (h1l) reciprocal space of a α′-P_3N_5 single crystal at 1 bar. For clarity only a few reflections are notated with their corresponding (hkl) index, although all visible reflections of the α′-P_3N_5 single crystal are encircled in blue. Other diffraction spots belong to distinct α′-P_3N_5 single crystal or the diamond anvils, while the continuous rings originate from the rhenium gasket.

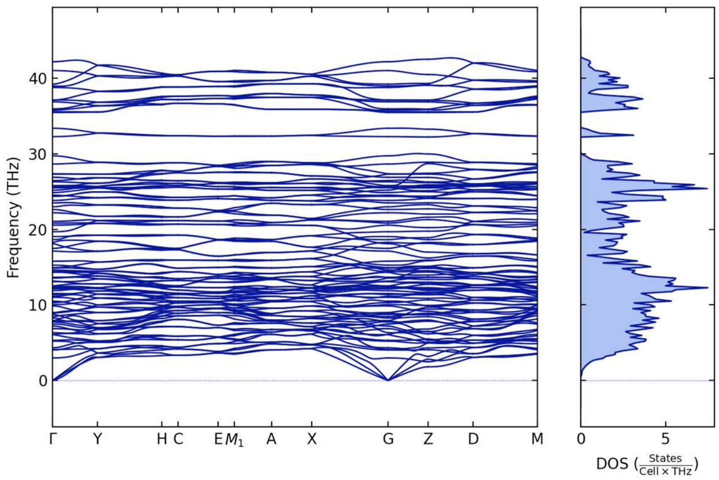

Figure S7: Calculated phonon band structure for α′-P_3N_5 at ambient pressure.

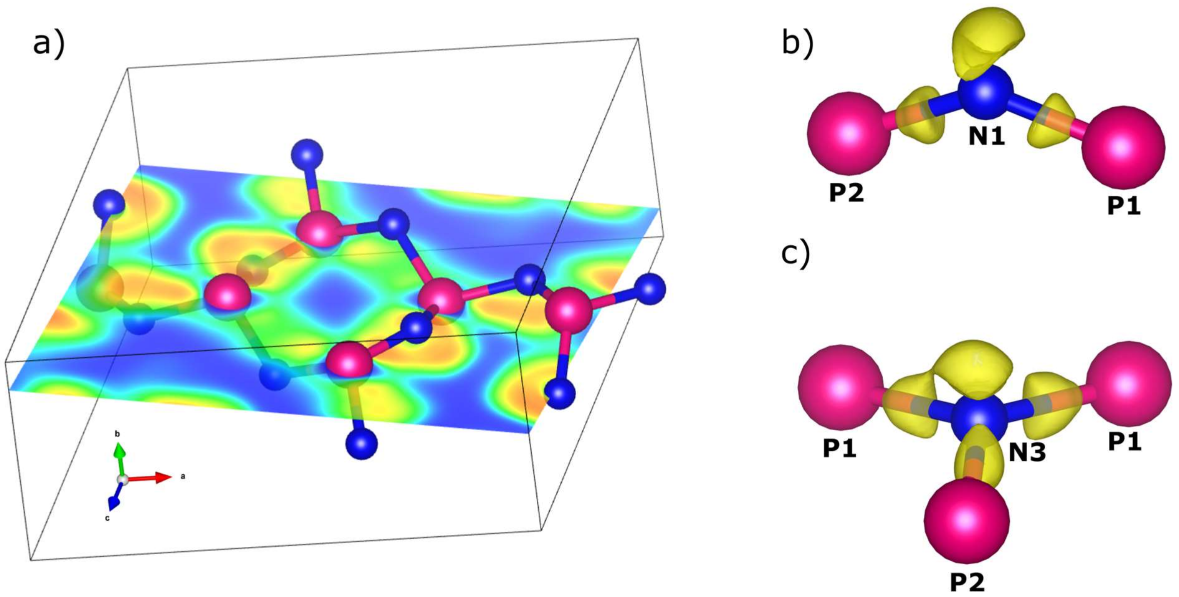

Figure S8: a) ELF slice of α′-P_3N_5 at 1 bar through the phosphorus atoms. b) and c) are the electronic isosurfaces drawn at 0.85 $e^-/Å^3$ for the N1 and N3 atoms, respectively. The N1 atom is a $N^{[2]}$ center while N3 is an $N^{[3]}$ center. The P-N bonds are seen to be polar covalent. The pink and blue spheres represent, respectively, phosphorus and nitrogen atoms.

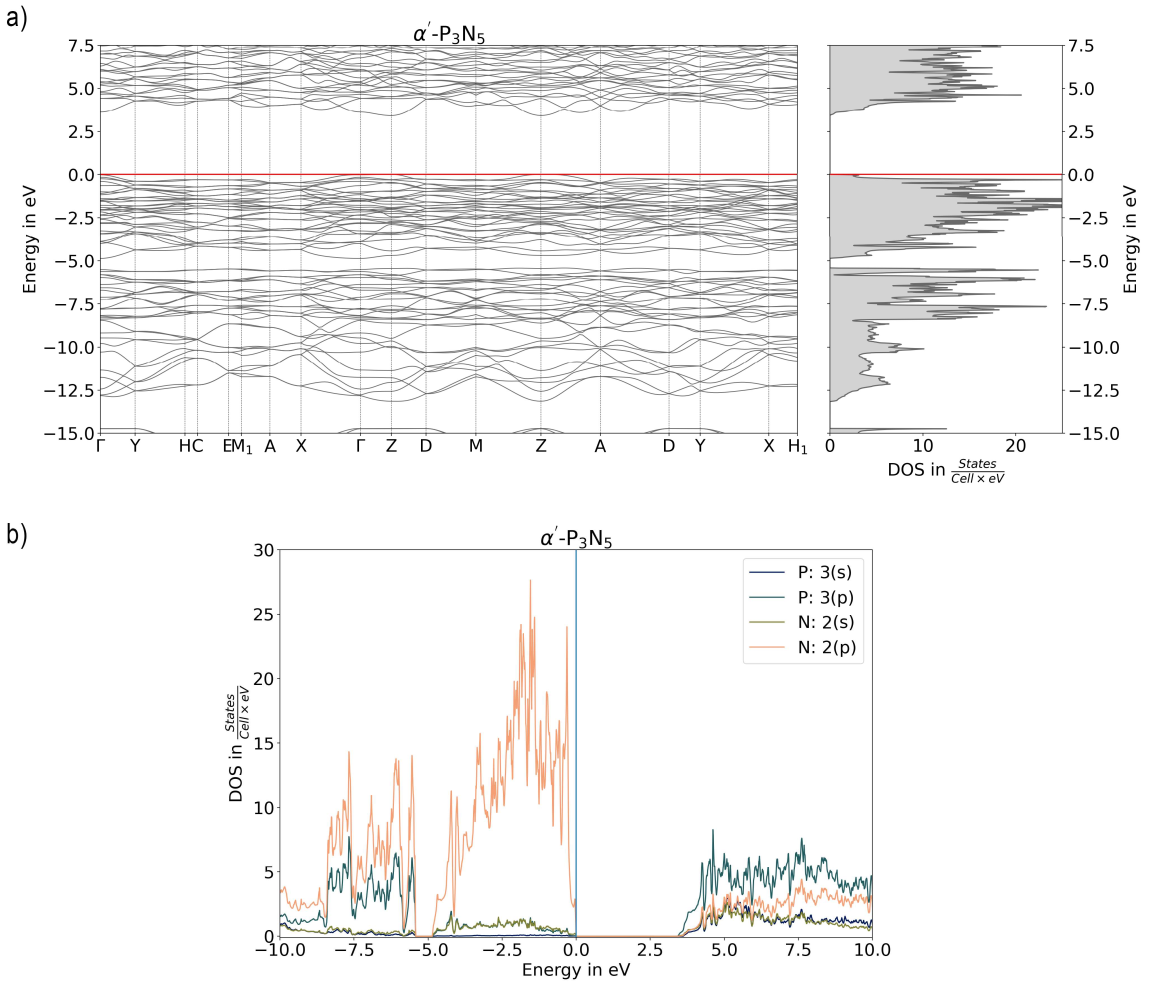

Figure S9: Calculated electronic structure of α′-P_3N_5 at ambient pressure: a) electronic band structure and total electronic density of states and b) orbital projected electronic density of states.

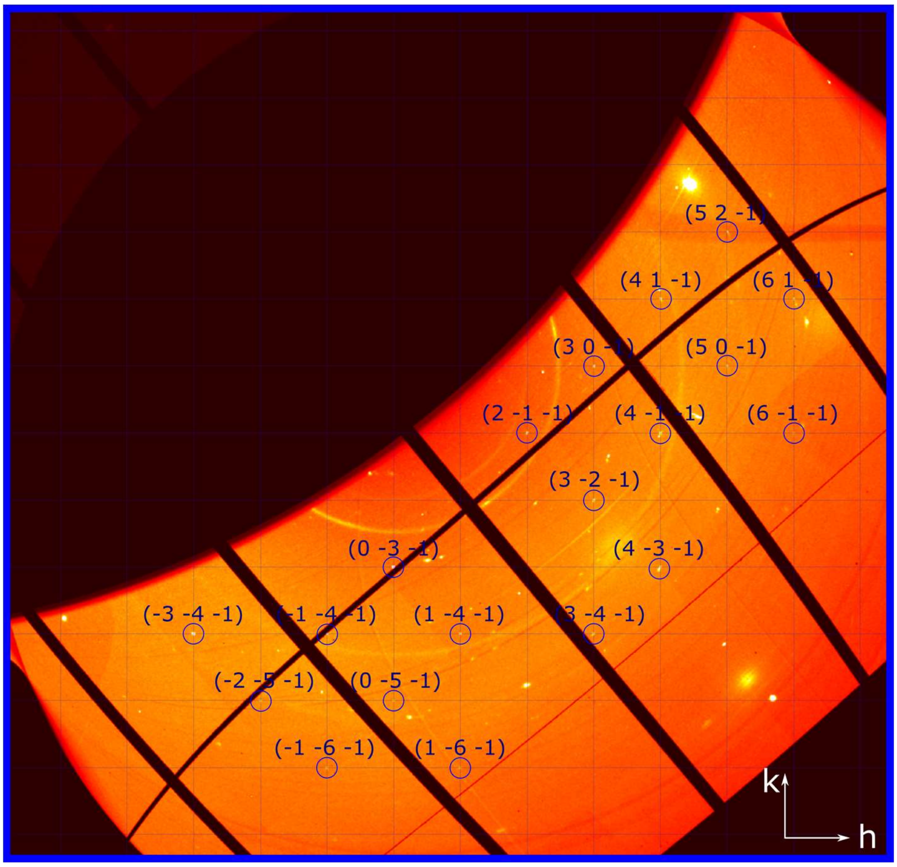

Figure S10: Slice of the (hk-1) reciprocal space of a PN_2 single crystal at 137 GPa. All visible reflections of the PN_2 single crystal are encircled in blue and are notated with their corresponding (hkl) index. Other diffraction spots belong to distinct PN_2 single crystal or the diamond anvils, while the continuous rings originate from the rhenium gasket.

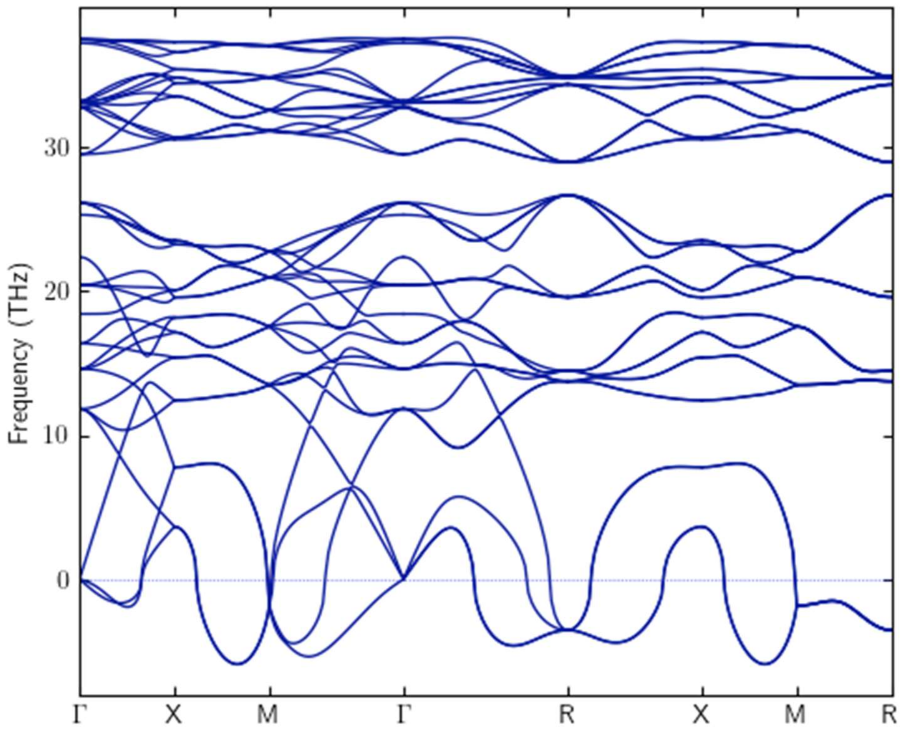

Figure S11: Calculated phonon band structure for the DFT-relaxed PN_2 at 137 GPa, with N-N distances of about 1.9 Å.

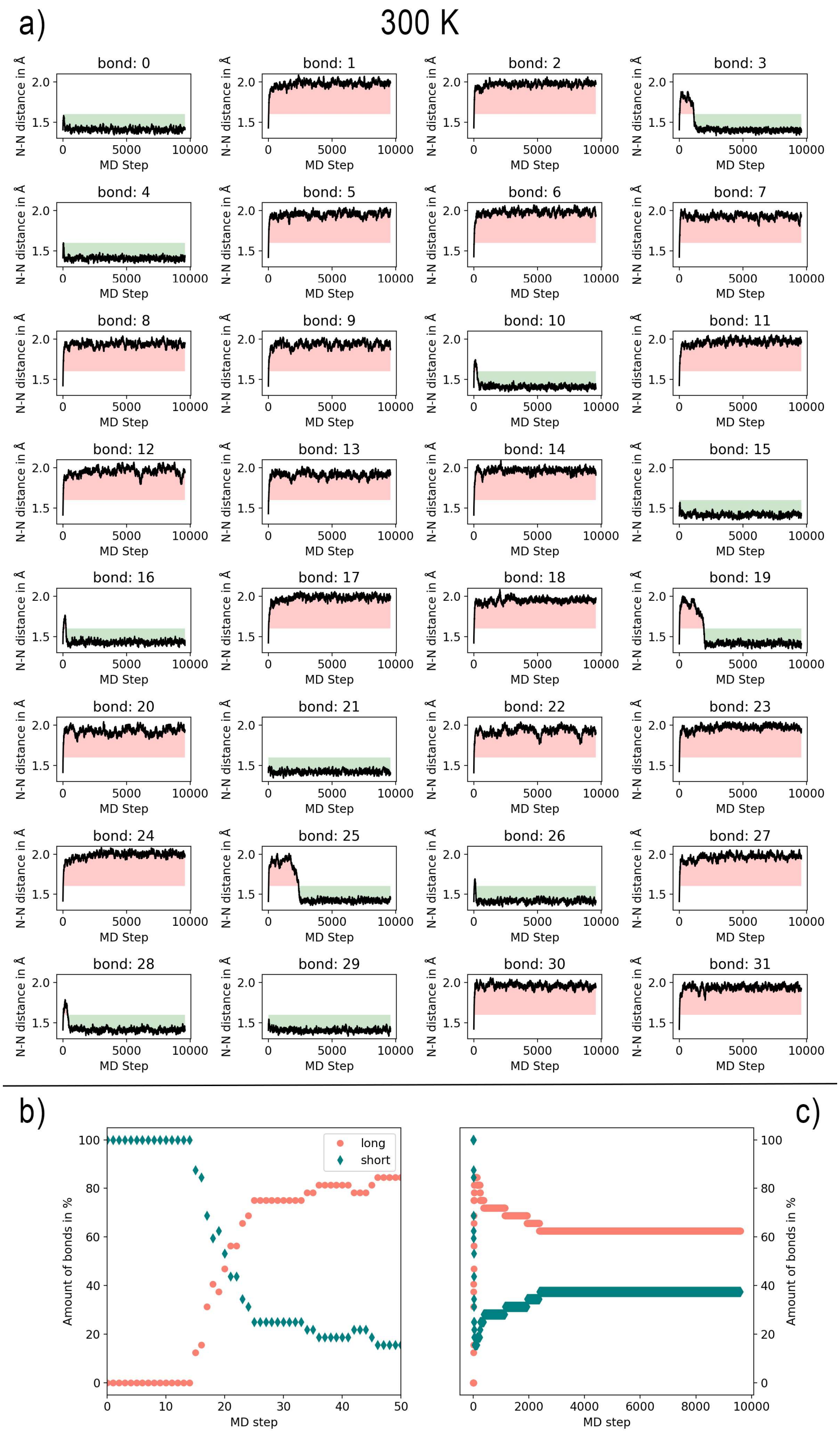

Figure S12: a) Bond length of the N-N distances connecting the PN_6 octahedra for 9600 MD steps of 0.9697 fs at 300 K starting from the experimental structure where all distances are ~1.4 Å. When colored green, the N-N distance is below 1.6 Å, and red when above this value, respectively suggesting bonding and the lack of bonding. b) and c) Percentage of long (>1.6 Å) and short (< 1.6 Å) N-N distances for the first 50 steps and the full simulation.

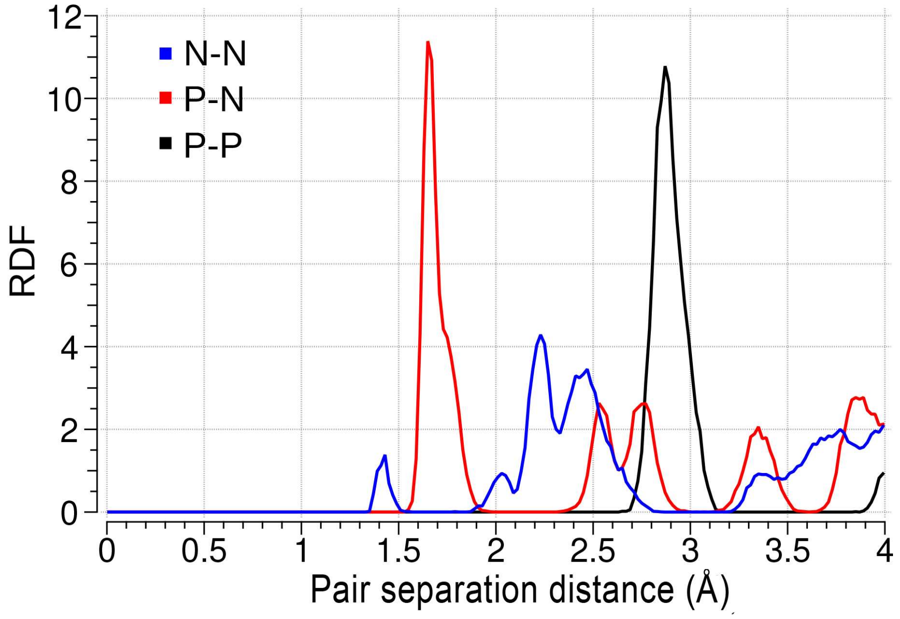

Figure S13: Radial distribution function of PN_2 calculated from the classical MD (192 atoms) at 300 K and 137 GPa.

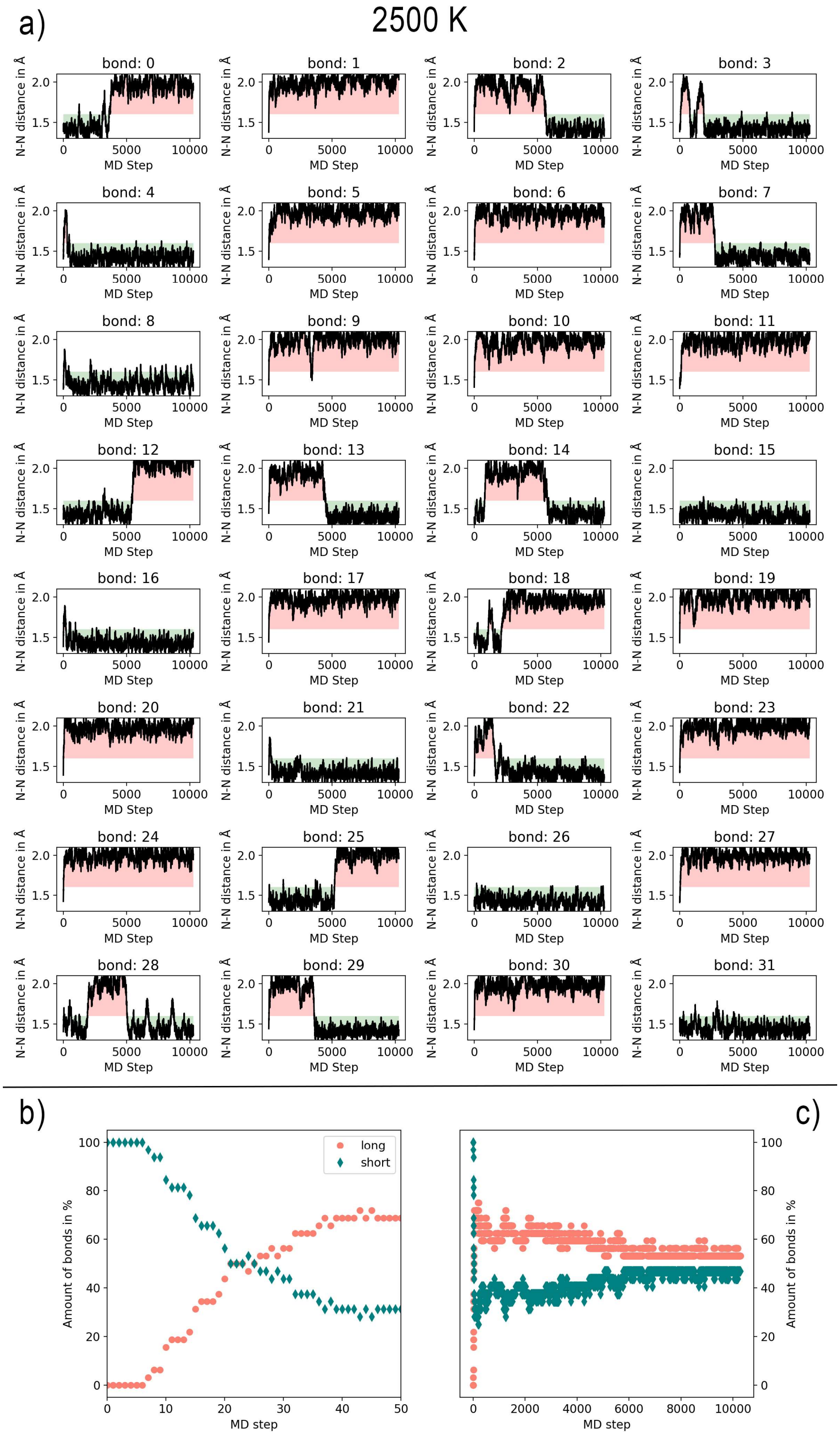

Figure S14: a) Bond length of the N-N distances connecting the PN_6 octahedra for 10200 MD steps of 0.9697 fs at 2500 K starting from the experimental structure where all distances are ~1.4 Å. When colored green, the N-N distance is below 1.6 Å, and red when above this value, respectively suggesting bonding and the lack of bonding. b) and c) Percentage of long (>1.6 Å) and short (< 1.6 Å) N-N distances for the first 50 steps and the full simulation.

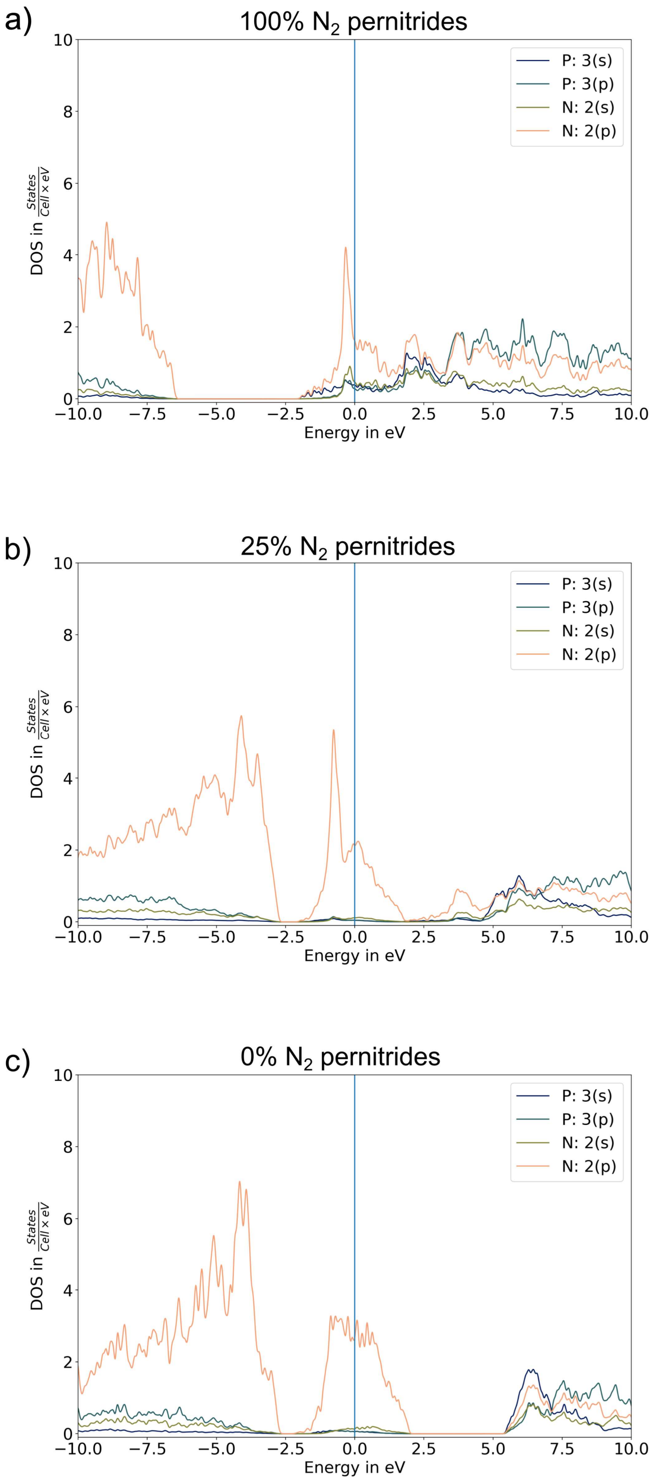

Figure S15: Projected electronic density of states for the PN_2 compound with a) all nitrogen atoms forming pernitride dimers (141 GPa), b) only 25% of all nitrogen atoms forming pernitride dimers (137 GPa), and c) no nitrogen atom forming pernitride dimers (137 GPa, fully relaxed). In all cases, PN_2 is found to be a metal.

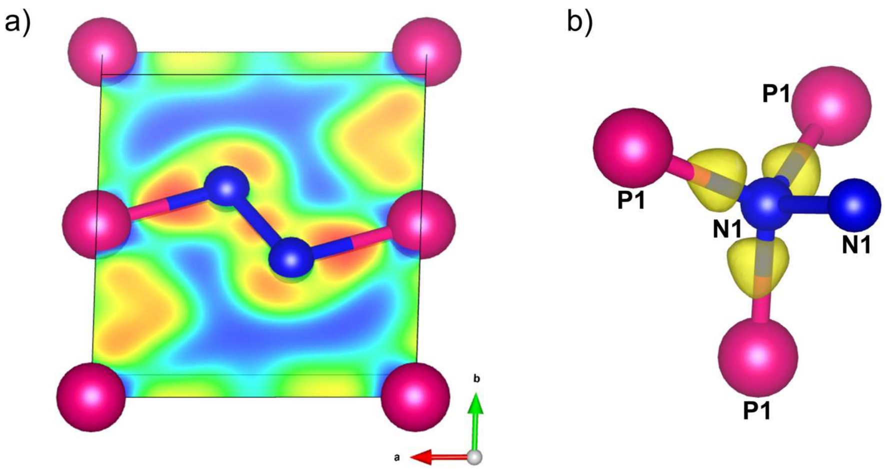

Figure S16: Electronic DFT calculations of PN_2 with 25% nitrogen atoms forming a N-N bond at 137 GPa. a) ELF of PN_2 with the electron localization corresponding to the P-N and the N-N bonds clearly visible. b) Electronic isosurface for 0.85 e^-/Å^3. The polar covalent P-N bond can be deduced from the conical shape of the isosurface. The electronic density between nitrogen atoms is hidden by the drawn bond. The pink and blue spheres represent, respectively, phosphorus and nitrogen atoms.

Table S1: Crystallographic data for δ-P_3N_5 at 72 GPa obtained by single-crystal X-ray diffraction. Some parameters have both the experimental and the calculated value. The crystallographic data has been submitted under the deposition number CSD 2178817.

	**δ-P_3N_5**			
	**Exp.**		**Calc.**	
Pressure (GPa)	72		72	
Space group, #	*C*2/*c*, 15		*C*2/*c*, 15	
Z	4		4	
a (Å)	8.418(5)		8.480	
b (Å)	4.325(6)		4.354	
c (Å)	6.040(4)		6.131	
β (°)	110.96(7)		110.94	
V ($Å^3$)	205.4(4)		211.4	
Refinement details				
Wavelength (λ, Å)	0.29521			
μ (mm^{-1})	0.259			
# measured/independent reflections (I ≥ 3σ)	345 / 202 (153)			
$(\sin θ/λ)_{max}$ ($Å^{-1}$)	0.867			
R_{int} (%)	3.14			
R_1 (%)	6.86			
wR_2 (%)	8.11			
R_1 (all data, %)	8.16			
wR_2 (all data, %)	8.19			
Goodness of fit	3.24			
No. of parameters	16			
$Δρ_{min}$, $Δρ_{max}$ ($eÅ^{-3}$)	-0.87, 1.14			
**Atomic positions**				

Atom	Wyckoff position	Fractional atomic coordinates (x; y; z)		U_{iso} ($Å^2$)
		**Exp.**	**Calc.**	
P1	8f	0.3667(2); 0.0097(3); 0.2815(4)	0.3681; 0.0096; 0.2830	0.0040(5)
P2	4a	1/2; 1/2; 1/2	1/2; 1/2; 1/2	0.0041(6)
N1	4e	1/2; 0.2984(15); 1/4	1/2; 0.2999; 1/4	0.0044(13)
N2	8f	0.4117(8); 0.1637(11); 0.0529(13)	0.4132; 0.1641; 0.0534	0.0052(9)
N3	8f	0.3127(7); 0.3476(11); 0.3537(13)	0.3102; 0.3469; 0.3510	0.0027(9)

Table S2: Crystallographic data for δ-P_3N_5 at 118 GPa obtained by single-crystal X-ray diffraction. The crystallographic data has been submitted under the deposition number CSD 2178818.

		**δ-P_3N_5**	
		**Exp.**	
Pressure (GPa)		118	
Space group, #		*C2/c*, 15	
Z		4	
a (Å)		8.237(18)	
b (Å)		4.251(2)	
c (Å)		5.931(12)	
β (°)		109.6(3)	
V (Å^3)		195.6(7)	
Refinement details			
Wavelength (λ, Å)		0.29521	
μ (mm^{-1})		0.272	
# measured/independent reflections (I ≥ 3σ)		270 / 160 (92)	
$(\sin \theta/\lambda)_{max}$ (Å^{-1})		0.867	
R_{int} (%)		1.34	
R_1 (%)		9.18	
wR_2 (%)		10.18	
R_1 (all data, %)		13.46	
wR_2 (all data, %)		10.51	
Goodness of fit		3.90	
No. of parameters		16	
$\Delta\rho_{min}$, $\Delta\rho_{max}$ (eÅ^{-3})		-1.52, 1.69	
**Atomic positions**			
Atom	Wyckoff position	Fractional atomic coordinates (x; y; z)	U_{iso} (Å^2)
		**Exp.**	
P1	8f	0.3680(5) 0.0079(8) 0.2838(7)	0.0064(8)
P2	4a	1/2; 1/2; 1/2	0.0033(8)
N1	4e	1/2; 0.307(3); 1/4	0.005(2)
N2	8f	0.4123(18) 0.169(2) 0.053(2)	0.0039(16)
N3	8f	0.313(2) 0.343(2) 0.355(3)	0.0041(15)

Table S3: Crystallographic data for α′-P_3N_5 at 1 bar obtained by single-crystal X-ray diffraction. Some parameters have both the experimental and the calculated value. The crystallographic data has been submitted under the deposition number CSD 2178819.

		α′-P_3N_5		
		**Exp.**	**Calc.**	
Pressure		ambient	ambient	
Space group, #		$P2_1/c$	$P2_1/c$	
Z		4	4	
a (Å)		9.2557(6)	9.3144	
b (Å)		4.6892(3)	4.7310	
c (Å)		8.2674(6)	8.3319	
β (°)		104.160(6)	104.089	
V (Å^3)		347.92(5)	356.11	
Refinement details				
Wavelength (λ, Å)		0.41015		
μ (mm^{-1})		0.327		
# measured/independent reflections (I ≥ 3σ)		994 / 625 (354)		
(sin θ/λ)$_{max}$ (Å^{-1})		0.893		
R_{int} (%)		4.54		
R_1 (%)		9.80		
wR_2 (%)		9.25		
R_1 (all data, %)		14.05		
wR_2 (all data, %)		9.50		
Goodness of fit		3.58		
No. of parameters		48		
$\Delta\rho_{min}$, $\Delta\rho_{max}$ (eÅ^{-3})		-1.24, 0.98		
**Atomic positions**				
Atom	Wyckoff position	Fractional atomic coordinates (x; y; z)		U_{iso} (Å^2)
		**Exp.**	**Calc.**	
P1	4e	0.5969(3); 0.0609(8); 0.2702(4)	0.5966; 0.0578; 0.2706	0.0205(11)
P2	4e	0.3016(3); 0.0829(8); 0.0296(4)	0.3016; 0.08331; 0.0288	0.0197(11)
P3	4e	0.0003(3); 0.0933(9); 0.1501(4)	0.0007; 0.0888; 0.1495	0.0241(12)
N1	4e	0.6949(9); 0.042(2); 0.1484(12)	0.6974; 0.042; 0.1454	0.021(2)
N2	4e	0.1281(10); 0.012(2); 0.0375(13)	0.1263; 0.008; 0.0339	0.020(2)
N3	4e	0.5765(8); 0.412(2); 0.3157(11)	0.5756; 0.410; 0.3126	0.0155(17)
N4	4e	0.3435(8); 0.403(2); 0.0588(11)	0.3449; 0.593; 0.0561	0.020(2)
N5	4e	0.0119(8); 0.917(3); 0.3147(12)	0.0120; 0.915; 0.3149	0.023(2)

Table S4: Lattice parameters, unit cell volume and number of correlated reflections for δ-P_3N_5 single-crystals during sample decompression.

Pressure (GPa)	*a* (Å)	*b* (Å)	*c* (Å)	*β* (°)	*V* (Å^3)	# reflections
85.7	8.370(5)	4.312(6)	6.042(4)	110.73(7)	203.9(3)	267
76.2	8.473(5)	4.3099(9)	6.053(2)	110.12(5)	207.53(15)	207
65.0	8.4897(17)	4.3431(3)	6.106(1)	110.040(18)	211.52(6)	172
56.3	8.5295(16)	4.3761(5)	6.142(4)	110.72(4)	214.43(15)	197
42.7	8.6470(15)	4.400(3)	6.1996(13)	110.795(19)	220.48(15)	182
33.6	8.674(4)	4.4286(6)	6.255(8)	110.73(9)	224.7(3)	151
23.7	8.724(4)	4.469(1)	6.323(13)	110.99(12)	230.2(5)	174
11.8	8.8128(19)	4.5108(5)	6.396(4)	110.85(4)	237.59(15)	236
7.0	8.8352(14)	4.5392(5)	6.4589(6)	110.899(14)	241.99(5)	172

Table S5: Crystallographic data for PN_2 at 134 GPa. The crystallographic data has been submitted under the deposition number CSD 2178820.

		**PN_2**	
		**Exp.**	
Pressure (GPa)		134	
Space group, #		*Pa*-3	
Z		4	
a (Å)		4.0127(14)	
V (Å^3)		64.61(4)	
Refinement details			
Wavelength (λ, Å)		0.2852	
μ (mm^{-1})		0.266	
# measured/independent reflections ($I \geq 3\sigma$)		253 / 93 (50)	
$(\sin \theta/\lambda)_{max}$ (Å^{-1})		1.182	
R_{int} (%)		3.52	
R_1 (%)		6.59	
wR_2 (%)		6.73	
R_1 (all data, %)		10.54	
wR_2 (all data, %)		6.84	
Goodness of fit		2.64	
No. of parameters		6	
$\Delta\rho_{min}$, $\Delta\rho_{max}$ (eÅ^{-3})		-0.95, 1.28	
**Atomic positions**			
Atom	Wyckoff position	Fractional atomic coordinates (x; y; z)	U_{iso} (Å^2)
		**Exp.**	
P1	4a	0; 0; 0	0.0139(2)
N1	8c	0.3981(7); 0.3981(7); 0.3981(7)	0.0120(2)

Table S6: Crystallographic data for PN_2 at 137 GPa. Some parameters have both the experimental and the calculated value. The calculated structure did not allow for the refinement of the atomic positions, but solely the unit cell volume. The crystallographic data has been submitted under the deposition number CSD 2178821.

	**PN_2**
	**Exp.**
Pressure (GPa)	137
Space group, #	*Pa*-3
Z	4
a (Å)	4.0036(6)
V (Å^3)	64.173(17)
Refinement details	
Wavelength (λ, Å)	0.3738
μ (mm^{-1})	0.474
# measured/independent reflections (I ≥ 3σ)	108 / 40 (25)
(sin θ/λ)$_{max}$ (Å^{-1})	0.943
R_{int} (%)	2.71
R_1 (%)	4.87
wR_2 (%)	6.21
R_1 (all data, %)	6.67
wR_2 (all data, %)	6.27
Goodness of fit	3.15
No. of parameters	6
$\Delta\rho_{min}$, $\Delta\rho_{max}$ (eÅ^{-3})	-1.1, 1.1

**Atomic positions**			
Atom	Wyckoff position	Fractional atomic coordinates (x; y; z)	U_{iso} (Å^2)
		**Exp.**	
P1	4a	0; 0; 0	0.0142(5)
N1	8c	0.3972(13); 0.3972(13); 0.3972(13)	0.0101(8)

References

[1] I. Kantor, V. Prakapenka, A. Kantor, P. Dera, A. Kurnosov, S. Sinogeikin, N. Dubrovinskaia, L. Dubrovinsky, *Rev. Sci. Instrum.* **2012**, *83*, 125102.

[2] A. Kurnosov, I. Kantor, T. Boffa-Ballaran, S. Lindhardt, L. Dubrovinsky, A. Kuznetsov, B. H. Zehnder, *Rev. Sci. Instrum.* **2008**, *79*.

[3] Y. Akahama, H. Kawamura, *J. Phys. Conf. Ser.* **2010**, *215*, 012195.

[4] T. Fedotenko, L. Dubrovinsky, G. Aprilis, E. Koemets, A. Snigirev, I. Snigireva, A. Barannikov, P. Ershov, F. Cova, M. Hanfland, N. Dubrovinskaia, *Rev. Sci. Instrum.* **2019**, *90*, 104501.

[5] Rigaku Oxford Diffraction, **2015**.

[6] V. Petrícek, M. Dušek, L. Palatinus, *Zeitschrift fur Krist.* **2014**, *229*, 345–352.

[7] O. V. Dolomanov, L. J. Bourhis, R. J. Gildea, J. A. K. Howard, H. Puschmann, *J. Appl. Crystallogr.* **2009**, *42*, 339–341.

[8] D. Laniel, M. Bykov, T. Fedotenko, A. V. Ponomareva, I. A. Abrikosov, K. Glazyrin, V. Svitlyk, L. Dubrovinsky, N. Dubrovinskaia, *Inorg. Chem.* **2019**, *58*, 9195–9204.

[9] D. Laniel, B. Winkler, E. Koemets, T. Fedotenko, S. Chariton, V. Milman, K. Glazyrin, V. Prakapenka, L. Dubrovinsky, N. Dubrovinskaia, *IUCrJ* **2021**, *8*, 208–214.

[10] D. Laniel, A. A. Aslandukova, A. N. Aslandukov, T. Fedotenko, S. Chariton, K. Glazyrin, V. B. Prakapenka, L. S. Dubrovinsky, N. Dubrovinskaia, *Inorg. Chem.* **2021**, *60*, 14594–14601.

[11] D. Laniel, T. Fedotenko, B. Winkler, A. Aslandukova, A. Aslandukov, G. Aprilis, S. Chariton, V. Milman, V. Prakapenka, L. Dubrovinsky, N. Dubrovinskaia, *J. Chem. Phys.* **2022**, *156*, 044503.

[12] E. Bykova, Single-Crystal X-Ray Diffraction at Extreme Conditions in Mineral Physics and Material Sciences, University of Bayreuth, **2015**.

[13] C. Prescher, V. B. Prakapenka, *High Press. Res.* **2015**, *35*, 223–230.

[14] D. . Frost, B. . Poe, R. . Trønnes, C. Liebske, A. Duba, D. . Rubie, *Phys. Earth Planet. Inter.* **2004**, *143–144*, 507–514.

[15] P. Giannozzi, S. Baroni, N. Bonini, M. Calandra, R. Car, C. Cavazzoni, D. Ceresoli, G. L. Chiarotti, M. Cococcioni, I. Dabo, A. Dal Corso, S. de Gironcoli, S. Fabris, G. Fratesi, R. Gebauer, U. Gerstmann, C. Gougoussis, A. Kokalj, M. Lazzeri, L. Martin-Samos, N. Marzari, F. Mauri, R. Mazzarello, S. Paolini, A. Pasquarello, L. Paulatto, C. Sbraccia, S. Scandolo, G. Sclauzero, A. P. Seitsonen, A. Smogunov, P. Umari, R. M. Wentzcovitch, *J. Phys. Condens. Matter* **2009**, *21*, 395502.

[16] P. Giannozzi, O. Andreussi, T. Brumme, O. Bunau, M. Buongiorno Nardelli, M. Calandra, R. Car, C. Cavazzoni, D. Ceresoli, M. Cococcioni, N. Colonna, I. Carnimeo, A. Dal Corso, S. de Gironcoli, P. Delugas, R. A. DiStasio, A. Ferretti, A. Floris, G. Fratesi, G. Fugallo, R. Gebauer, U. Gerstmann, F. Giustino, T. Gorni, J. Jia, M. Kawamura, H.-Y. Ko, A. Kokalj, E. Küçükbenli, M. Lazzeri, M. Marsili, N. Marzari, F. Mauri, N. L. Nguyen, H.-V. Nguyen, A. Otero-de-la-Roza, L. Paulatto, S. Poncé, D. Rocca, R. Sabatini, B. Santra, M. Schlipf, A. P. Seitsonen, A. Smogunov, I. Timrov, T. Thonhauser, P. Umari, N. Vast, X. Wu, S. Baroni, *J. Phys. Condens. Matter* **2017**, *29*, 465901.

[17] P. Giannozzi, O. Baseggio, P. Bonfà, D. Brunato, R. Car, I. Carnimeo, C. Cavazzoni, S. de Gironcoli, P. Delugas, F. Ferrari Ruffino, A. Ferretti, N. Marzari, I. Timrov, A. Urru, S. Baroni, *J.*

Chem. Phys. **2020**, *152*, 154105.

[18] P. E. Blöchl, *Phys. Rev. B* **1994**, *50*, 17953–17979.

[19] S. Grimme, J. Antony, S. Ehrlich, H. Krieg, *J. Chem. Phys.* **2010**, *132*, 154104.

[20] H. J. Monkhorst, J. D. Pack, *Phys. Rev. B* **1976**, *13*, 5188–5192.

[21] A. Togo, I. Tanaka, *Scr. Mater.* **2015**, *108*, 1–5.

[22] A. V Shapeev, *Multiscale Model. Simul.* **2016**, *14*, 1153–1173.

[23] I. S. Novikov, K. Gubaev, E. V Podryabinkin, A. V Shapeev, *Mach. Learn. Sci. Technol.* **2021**, *2*, 025002.

[24] G. Kresse, J. Hafner, *Phys. Rev. B* **1993**, *47*, 558–561.

[25] G. Kresse, J. Hafner, *Phys. Rev. B* **1994**, *49*, 14251–14269.

[26] G. Kresse, J. Furthmüller, *Comput. Mater. Sci.* **1996**, *6*, 15–50.

[27] G. Kresse, J. Furthmüller, *Phys. Rev. B* **1996**, *54*, 11169–11186.

[28] A. P. Thompson, H. M. Aktulga, R. Berger, D. S. Bolintineanu, W. M. Brown, P. S. Crozier, P. J. in 't Veld, A. Kohlmeyer, S. G. Moore, T. D. Nguyen, R. Shan, M. J. Stevens, J. Tranchida, C. Trott, S. J. Plimpton, *Comput. Phys. Commun.* **2022**, *271*, 108171.

[29] J. Heyd, G. E. Scuseria, M. Ernzerhof, *J. Chem. Phys.* **2003**, *118*, 8207–8215.

[30] K. Niwa, Y. Iijima, M. Ukita, R. Toda, K. Toyoura, T. Sasaki, K. Matsunaga, N. A. Gaida, M. Hasegawa, *J. Raman Spectrosc.* **2021**, *52*, 1064–1072.
